# Plant sizes and shapes above and belowground and their interactions with climate

**DOI:** 10.1111/nph.18031

**Published:** 2022-03-08

**Authors:** Shersingh Joseph Tumber‐Dávila, H. Jochen Schenk, Enzai Du, Robert B. Jackson

**Affiliations:** ^1^ Department of Earth System Science Stanford University 473 Via Ortega Stanford CA 94305 USA; ^2^ Harvard Forest Harvard University 324 N Main St Petersham MA 01366 USA; ^3^ Department of Biological Science California State University Fullerton 800 North State College Blvd Fullerton CA 92831 USA; ^4^ Faculty of Geographical Science Beijing Normal University 19 Xinjiekouwai Street Beijing 100875 China; ^5^ Woods Institute for the Environment Stanford University 473 Via Ortega Stanford CA 94305 USA; ^6^ Precourt Institute for Energy Stanford University 473 Via Ortega Stanford CA 94305 USA

**Keywords:** allometry, lateral root spread, plant shape, plant–environment interactions, root systems, rooting depth, shoot height, shoot width

## Abstract

- Although the above and belowground sizes and shapes of plants strongly influence plant competition, community structure, and plant–environment interactions, plant sizes and shapes remain poorly characterized across climate regimes. We investigated relationships among shoot and root system size and climate.
- We assembled and analyzed, to our knowledge, the largest global database describing the maximum rooting depth, lateral spread, and shoot size of terrestrial plants – more than doubling the Root Systems of Individual Plants database to 5647 observations.
- Water availability and growth form greatly influence shoot size, and rooting depth is primarily influenced by temperature seasonality. Shoot size is the strongest predictor of lateral spread, with root system diameter being two times wider than shoot width on average for woody plants.
- Shoot size covaries strongly with rooting system size; however, the geometries of plants differ considerably across climates, with woody plants in more arid climates having shorter shoots, but deeper, narrower root systems. Additionally, estimates of the depth and lateral spread of plant root systems are likely underestimated at the global scale.

## Abstract 2

See also the Commentary on this article by Kattge, **235**: 821–823.

## Introduction

The vertical and horizontal extents of plants partly define plant architecture above and belowground (Lynch, 1995; Schenk & Jackson, 2002a; Hunt, 2016; Pawlik & Kasprzak, 2017). Plant architecture, the three‐dimensional organization of the plant body (Reinhardt & Kuhlemeier, 2002), is plastic; plants compensate for resource limitations by altering allocation among above and belowground organs to optimize growth, survival, and reproduction (Poorter *et al*., 2012; Díaz *et al*., 2016). To understand plant responses to changes in resource availability and climate (Dybzinski *et al*., 2011; Farrior *et al*., 2015), several global studies have examined plant biomass partitioning across climates (Cheng & Niklas, 2006; Mokany *et al*., 2006; Reich *et al*., 2014). However, the vertical and horizontal extents of plants have traditionally been ignored, despite the fact that plants with similar biomass allometries may have different dimensions. In this study we seek to understand how the maxima of plant extents respond to climate through changes among shoot height and width and rooting depth and spread.

Understanding the relationships between the size of plants above and belowground will improve our knowledge of plant form and function. For example, the global spectrum of plant form and function (plant economic spectrum; PES), proposed by Díaz *et al*. (2016), posits that the size of plants and their organs represents the first major dimension of the PES. Consequently, most PES studies have focused on leaf, seed, and stem traits; however, these studies have typically used only shoot height to represent overall plant size (Verbeeck *et al*., 2019). Additionally, root traits, such as maximum depth and spread, considered to be an important missing link, have mostly been excluded from such analyses due to a scarcity of data (Joswig *et al*., 2022). When root traits have been included in studies of the global spectrum of plant form and function, the focus has usually been on fine root traits, not root system size traits, such as maximum depth and spread (Carmona *et al*., 2021).

Variation in belowground plant traits remains poorly quantified compared with shoot traits (Jackson *et al*., 1996; Vogt *et al*., 1996; Norby & Jackson, 2000; Reich, 2014; Iversen & McCormack, 2021). The size and shape of root systems rely, first, on resource demand of the plant (for water and nutrients), depending on overall plant size and growth strategy (Jackson *et al*., 2000; Enquist & Niklas, 2002; Niklas & Enquist, 2002; Poorter *et al*., 2012); second, on resource availability belowground (Poorter & Nagel, 2000; Schenk, 2008a); third, on soil constraints, such as horizons, bedrock, hardpans, and groundwater tables (Brantley *et al*., 2017; Fan *et al*., 2017; Hasenmueller *et al*., 2017); and fourth, on the presence, size, and identity of competing root systems (Caldwell *et al*., 1985; Casper & Jackson, 1997; Schenk *et al*., 1999; Casper *et al*., 2003; Dannowski & Block, 2005; Schenk, 2006; van Noordwijk *et al*., 2015). The complexity of the belowground environment coupled with methodological challenges make quantifying plant–root–environment interactions difficult, especially in the field.

Furthermore, compiled data on root system size are scarce (Guerrero‐Ramírez *et al*., 2021). Although scarce, estimates of maximum rooting depth remain one of the most sought‐after plant traits, with 10% of the thousands of TRY plant‐trait database inquiries requesting maximum rooting depth data (Kattge *et al*., 2020). One reason for the demand of rooting depth data is that the depth and lateral placement of roots influences plant–soil interactions, thereby affecting element cycling, plant water uptake, and soil organic matter content (Jobbágy & Jackson, 2000; Poirier *et al*., 2018; Freschet *et al*., 2021b). Additionally, rooting depth is a key plant trait used by most terrestrial‐biosphere models to estimate plant water uptake (Warren *et al*., 2015; Stocker *et al*., 2021).

Maximum rooting depth has been evaluated through quantitative syntheses such as those of Schenk & Jackson (2002a) and Fan *et al*. (2017), which acknowledge many important earlier studies (e.g. Weaver, 1919; Phillips, 1963; Canadell *et al*., 1996). Deeper rooting has been found more often for plants limited by water availability (Freschet *et al*., 2021a). Relative to plant size, rooting depths increase with aridity and seasonality, and the deepest roots are often found where there is evaporative demand during dry seasons for water available deeper in the soil (Schenk & Jackson, 2005). Additionally, Fan *et al*. (2017) found that variations in the soil water profile caused by infiltration, drainage, and water table depth helped explain considerable variation in rooting depth. These maximum rooting depth syntheses have led to the following biome‐level characterizations: relatively shallow‐rooted ecosystems tend to be found in boreal and permafrost regions, wetlands, and land covered by annual plants, whereas relatively deeper roots are found in more arid, semi‐arid, and seasonally dry climates (Schenk & Jackson, 2005; Fan *et al*., 2017). In summary, the distribution of water belowground and the seasonal variation in the amount, location of – and demand for – water strongly affect the depth of plant roots.

Even rarer than rooting depth data are datasets of maximum lateral spread (Klimešová *et al*., 2018; Guerrero‐Ramírez *et al*., 2021). Lateral rooting extent is the maximum horizontal distance between roots and the base of the plant. The lateral extent of roots affects nutrient foraging (Cahill & McNickle, 2011; Giehl & von Wirén, 2014), shoot anchorage (Ennos, 1993; Schwarz *et al*., 2010), and competition (Casper & Jackson, 1997; Schenk *et al*., 1999; Casper *et al*., 2003; Schenk, 2006). Lateral rooting extent can also be an extremely plastic trait (Klimešová *et al*., 2018). Plants have been found to explore large volumes of soil; for example, grasses and trees in the Namib Desert have lateral root extents as great as 12 m and 50 m, respectively (Kutschera, 1997).

**Fig. 1:**
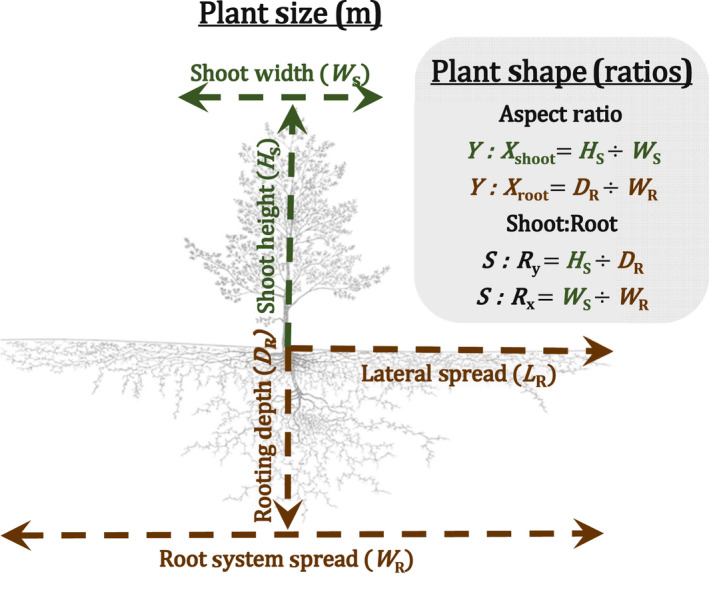
The main plant growth extents as defined in the Root Systems of Individual Plants (RSIP). The plant size measures, or the absolute extents, illustrate the maximum aboveground (in green – shoot width and shoot height) and belowground (in brown – rooting depth, lateral spread, and root system spread) extents in meters. The inset gray box shows the four plant shape ratios used to understand the dimensions or aspect ratio of the shoot (*Y* : *X*_shoot_) and the root system (*Y* : *X*_root_), and the above/belowground vertical (*S* : *R_y_*) and horizontal (*S* : *R_x_*) allometry. The tree outline was adapted from figure 115 of *Wurzelatlas mitteleuropäischer Waldbäume und Sträuche* (Kutschera & Lichtenegger, 2002).

**Fig. 2:**
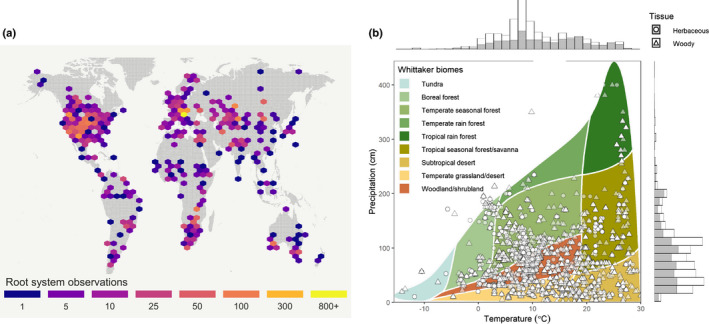
The (a) geographic and (b) climatic distributions of Root Systems of Individual Plants (RSIP) database records. (a) Global hexbin map showing the geographic distribution of RSIP observations, split into 50 hexagonal bins. (b) Whittaker plot of RSIP observations separated into woody (triangles) and herbaceous (circles) plants. The plot shows the distribution of biomes based on mean annual precipitation and temperature (as defined in the figure key), and how the RSIP observations fall within the climate space. The marginal histograms show the relative distribution of woody (white bars) and herbaceous (gray bars) plants across the axes.

To rectify the scarcity of root‐system size data, we assembled, to our knowledge, the largest global database describing the maximum rooting depth, lateral spread, and shoot size of terrestrial plants. The Root Systems of Individual Plants (RSIP) database was developed in 2002 to quantify the maximum depth *D*_R_ and lateral spread *L*_R_ of plant root systems (Schenk & Jackson, 2002a; Fig. 1; Supporting Information Fig. S1a). Here, we more than doubled the database to 5647 total observations across a broad range of terrestrial climates and geographies (Figs 2, S1c).

We use the expanded RSIP database to examine large‐scale patterns related to plant size and shape both above and belowground. Specifically, we seek to (1) characterize the root and shoot sizes of different plant functional types (PFTs), (2) understand how plant size, climate, and environment influence the vertical and horizontal extents of plants globally, (3) evaluate how plant dimensions shift above and belowground along climatic gradients, and (4) compare individual‐plant‐scale rooting depths to ecosystem‐scale rooting depths across biomes and climates.

## Materials and Methods

### Dataset

**Fig. 3:**
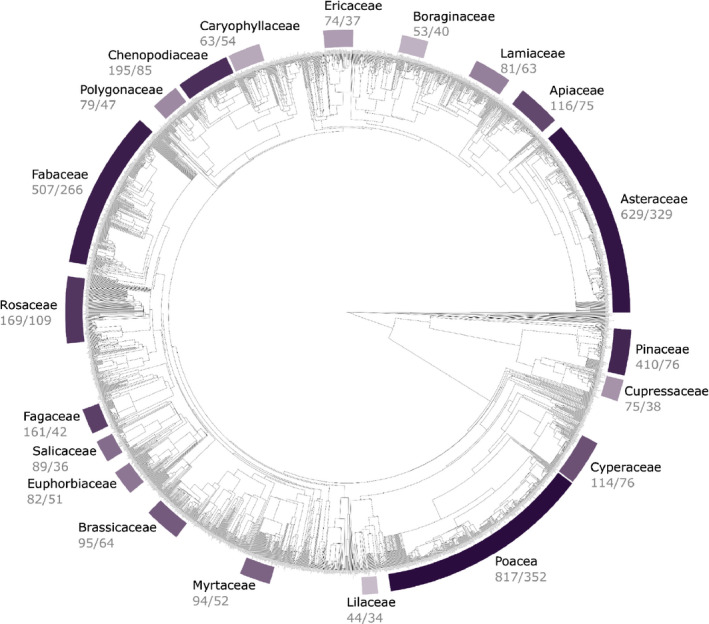
Phylogenetic tree of the 2989 species represented in the Root Systems of Individual Plants (RSIP). The highlighted plant families represent the 20 largest families in the RSIP based on the number of species represented (263 plant families overall). The 20 families represent 71% of all observations in the RSIP. The colors from light purple to dark purple represent the number of observations from each plant family. The labels show the plant family name, followed by the number of observations and the number of species (i.e. family no. of observations/no. of species).

**Fig. 4:**
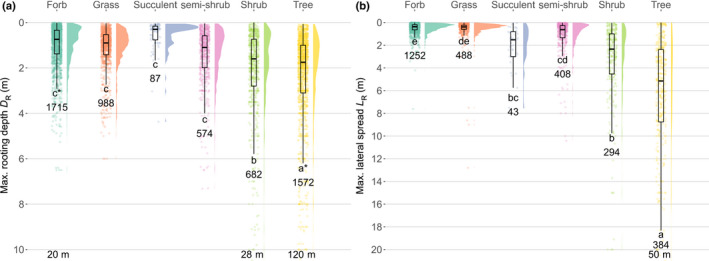
Raincloud plots for (a) maximum rooting depth *D*_R_ and (b) maximum lateral spread *L*_R_ across growth forms: forb, grass, succulent, semi‐shrub, shrub, and tree. The lowercase letters represent significantly different treatments for *D*_R_ and *L*_R_ across growth forms via Tukey’s honest significance difference tests. The horizontal lines in the boxplots represent the median values. The asterisk indicates the only situation where rooting depth relative to shoot volume differed between growth forms (i.e. the relative depth of forbs was significantly greater than for trees). The number at the end of each whisker indicates the total number of observations for each growth form. The maximum values for growth forms exceeding the plot scales are shown at the bottom.

The RSIP dataset integrates observations of the vertical and horizontal extents of individual plants with data for other plant traits. The RSIP data come from published observations of maximum plant root system dimensions, 361 publications (Appendix A1), covering 2989 species from 263 plant families (Fig. 3). The first version of the RSIP (Fig. S1a; Schenk & Jackson, 2002a) included 1305 observations for water‐limited ecosystems, and second version (Fig. S1b; Schenk & Jackson, 2005) included 2449 observations across a broader range of climates. Our expanded RSIP, with 5647 total observations (Fig. S1c), includes a range of root and shoot sizes spanning more than four orders of magnitude (Fig. 4) across most of the Earth’s climates and environments (Fig. 2).

The RSIP entries are classified by physiology and functional traits (see Tables S1, S2 for a full list of RSIP variables), including six growth forms: forbs (30% of observations), grasses (18%), semi‐shrubs (shrub species and suffrutescent forbs that rarely reach 1 m in height; 10%), shrubs (12%), stem succulents (2%), and trees (28%). We also record coarse‐scale information on the plant’s environment and location, such as biome, elevation, and spatial coordinates (see Tables S1, S2). There are, however, fine‐scale environmental parameters, such as soil traits, that cannot be accurately estimated based on the spatial coordinates for the RSIP entries.

The spatial coordinates allowed us to estimate related climate information, such as mean annual precipitation (MAP), when it was unavailable in the source literature. The estimated climate parameters came from WorldClim2, specifically 1 km spatial resolution climate surfaces for global land areas, providing historical (1970–2000) monthly and annual estimates of temperature and precipitation (Fick & Hijmans, 2017). Estimates for mean annual potential evapotranspiration (MAE) came from the Global Aridity Index and Potential Evapotranspiration Climate Database v.2 (Trabucco & Zomer, 2019). Nineteen additional bioclimatic variables were calculated following Fick & Hijmans (2017), providing long‐term metrics for precipitation and temperature seasonality (Table S1, BIO1‐19).

Bioclimatic variables allowed us to test how seasonality and climate affect the size of plants. Additionally, we calculated the seasonality of precipitation metric $S_{a}=\min\left[P_{\text{sur}},P_{\text{def}}\right]$ described in Schenk & Jackson (2005). To calculate *S*_a_, we used long‐term monthly average precipitation (Fick & Hijmans, 2017) and potential evapotranspiration (Trabucco & Zomer, 2019) to calculate the sum of the seasonal surplus *P*_sur_ or deficit *P*_def_ of water. See Table S1 for the equations and definitions for *S*_a_, *P*_def_, and *P*_sur_, along with a description of each of the climate metrics, growth extents, plant traits, and environmental metrics.

### Describing plant size

The RSIP contains measurements describing the maximum above and belowground dimensions of individual plants at the time of measurement. Maximum rooting depth *D*_R_ (*n* = 5633) is defined as the deepest soil depth reached by the roots of an individual plant (Fig. 1; Table 1). Two additional belowground dimensions in the database include lateral spread *L*_R_ (*n* = 2874), the maximum one‐sided horizontal distance from the stem of an individual plant reached by its roots (i.e. the radius), and root system width *W*_R_ (*n* = 1756), the maximum root system diameter, which is not always the same as 2 × *L*_R_ because most root systems are asymmetrical (Fig. 1). The main aboveground dimensions in the database are shoot height *H*_S_ (*n* = 2373) and shoot width *W*_S_ (*n* = 2074; Fig. 1), the maximum shoot diameter. Shoot volume *V*_S_ was estimated using an ellipsoid shape ($V_{S}\;\left(m^{3}\right)=\pi \times H_{S}\times W_{S}^{2}/6$). We excluded from the analyses of shoot width *W*_S_ and lateral spread *L*_R_ those observations from species known to have clonal, rhizomatous, or stoloniferous growth habits (*n* = 101), such as *Populus tremuloides* and *Poa pratensis*, so as not to give a misleading view of their functional morphology by only measuring the widths of individual ramets. The maximum dimensions of an individual plant at the time of excavation had to be directly measured to be included in the RSIP; observations were excluded from the RSIP if the sampling depth was less than the perceived max rooting depth, if allometric equations or other formulas were used to predict plant dimensions, or if the measurements were an aggregate of multiple observations and were not the dimensions of an individual plant.

**Table 1:** A list of commonly used abbreviations (see Supporting Information Table S1 for a list of all RSIP parameters).

Abbreviation	Explanation
Plant size	
*D*_R_	Maximum rooting depth of plant (m)
*L*_R_	Maximum lateral root spread, one‐sided (radius) linear distance from stem reached by roots (m)
*W*_R_	Rooting spread or diameter (m)
*H*_S_	Height of plant shoot (m)
*W*_S_	Width of plant shoot (m)
*V*_S_	Canopy volume, calculated using an ellipsoidal shape:
	$V_{S}\;\left(m^{3}\right)=\pi \times H_{S}\times W_{S}^{2}/6$
DBH	Stem diameter (diameter at breast height) of trees (cm)
Plant shape	
*Y* : *X*_shoot_	Aboveground dimensional aspect ratio (*Y* : *X*_shoot_ * = H*_S_/*W*_S_)
*Y* : *X*_root_	Belowground dimensional aspect ratio (*Y* : *X*_root_ =* D*_R_/*W*_R_)
*S* : *R_y_*	Vertical shoot : root ratio (*S* : *R_y_ = H*_S_/*D*_R_)
*S* : *R_x_*	Horizontal shoot : root ratio (*S* : *R_x_ = W*_S_/*W*_R_)
Climate	
MAP	Mean annual precipitation (m)
MAE	Mean annual potential evapotranspiration (m)
*A*_i_	Aridity index (*A*_i_ *= *MAP/MAE)
*S*_a_	Seasonality index or annual water storage index: *S*_a_ = min[*P*_sur_, *P*_def_]
Datasets	
RSIP	Root Systems of Individual Plants
RPGE	Root Profiles for Global Ecosystems (Schenk & Jackson, 2003)

### Phylogenetic analysis

To understand the importance of phylogeny on the main variables (*D*_R_, *L*_R_, *H*_S_, and *W*_S_), we calculated the phylogenetic signal using Pagel’s lambda (Pagel, 1997, 1999) and performed phylogenetically independent contrasts (PICs) between above and belowground plant extents and across the main climate metrics (MAE, MAP, aridity index *A*_i_, and *S*_a_). The phylogeny of RSIP observations was constructed using the v.phylomaker R package (Jin & Qian, 2019) with the GBOTB.extented mega‐tree (Zanne *et al*., 2014; Smith & Brown, 2018). The plant names were standardized using the *The Plant List* (2013; v.1.1; www.theplantlist.org/) to match the nomenclature present in the mega‐tree. Calculating Pagel’s lambda allowed us to estimate the phylogenetic signal of the plant trait in question, by estimating the magnitude by which shared phylogenetic history drives the trait distribution at the tips of the phylogeny (Freckleton *et al*., 2002). A lambda value of zero indicates no phylogenetic influence on plant traits, whereas a lambda value of one represents high phylogenetic signal. To calculate Pagel’s lambda and the log likelihood statistic we used the phytools::phylosig R function (Revell, 2012) to run 100 simulations for each of the plant extents separated into three groups: (1) all observations, (2) woody plants (trees, shrubs, and semi‐shrubs), and (3) herbaceous plants (forbs and grasses). We performed regressions of phylogenetically independent contrasts (Felsenstein, 1985) for each of the resolved phylogenies using the ape and stats R packages (Paradis & Schliep, 2019; R Core Team, 2020). Phylogenetic relatedness was calculated and used as a predictor variable in the random forest analysis (see next section) via an analysis of the phylogenetic pairwise distance between species using the ape package (Paradis & Schliep, 2019), as suggested in Bergmann *et al*. (2017).

### Evaluating variable importance for shoot and root extents

To determine factors influencing maximum root (*D*_R_ and *L*_R_) and shoot extents (*H*_S_ and *W*_S_), we estimated the importance of covariates using a random forest approach (Breiman, 2001). The list of covariates included aboveground plant traits and climate metrics (see Table S1 for a full list of RSIP parameters). The random forest models for belowground extents were run with (Fig. 6c,d see later) and without (Fig. 6a,b see later) aboveground size (*H*_S_, *W*_S_, and *V*_S_) as predictors; however, belowground extents (*D*_R_ and *L*_R_) were not used as predictors for *H*_S_ and *W*_S_.

For the random forest approach, we utilized the ranger package (Wright & Ziegler, 2017), which is an implementation of the original random forest (Breiman, 2001) suited for high‐dimensional data (Boehmke & Greenwell, 2020). We split the RSIP dataset using stratified sampling into a model training subset containing 70% of the entries and a model testing subset using the rsample package (Silge *et al*., 2021). Because random forests cannot handle missing values, we used the missranger package (Mayer, 2019) to impute missing values through a nonparametric approach for mixed‐type data using chains of random forests (Stekhoven & Buhlmann, 2012). The training data were used to adjust the random forest model using a hyperparameter grid to search for the optimal parameter values, resulting in the greatest reduction in root‐mean‐square error (Probst *et al*., 2019). The hyperparameter tuning resulted in an average 4% improvement compared with the baseline model. The random forest model was then rerun using the selected hyperparameters to calculate the permutation‐based variable importance for each predictor. We chose the permutation‐based method because it is not biased towards variables with high cardinality (Strobl *et al*., 2008), such as for many climate variables. Although the permutation‐based approach is more computationally intensive (because of the constant shuffling of features across the decision trees), it is generally a more accurate method than the standard mean‐decrease‐in‐impurity importance (Strobl *et al*., 2007).

Additionally, we sought to determine how plant size differed across categorical variables such as plant characteristics and growth form (Table 2). Significant differences between the plant extents of categorical parameters were tested using ANOVA and *post hoc* Tukey honest significant difference tests (de Mendiburu, 2021).

**Table 2:** Mean belowground (rooting depth (*D*_R_), lateral spread (*L*_R_), shoot height (*H*_S_) and shoot width (*W*_S_)) extents across plant traits.

	Belowground extents (m)				Aboveground extents (m)			
	*D*_R_		*L*_R_		*H*_S_		*W*_S_	
	Mean	SD and group	Mean	SD and group	Mean	SD and group	Mean	SD and group
Growth form								
Forb	1.02	1.04c	0.51	0.51e	0.36	0.34c	0.34	0.37c
Grass	1.14	0.93c	0.55	0.94de	0.55	0.53bc	0.35	0.85c
Semi‐shrub	1.42	1.2c	1.07	1.37cd	0.33	0.24c	0.53	0.55c
Shrub	2.36	2.85b	3.33	3.46b	1.47	1.5b	1.48	1.68b
Succulent	0.56	0.68c	2.22	1.93bc	0.61	0.64bc	0.78	0.55bc
Tree	3.64	7.69a	7.04	7a	8.07	9.12a	3.25	4.42a
Lifespan								
Annual	0.76	0.6b*	0.4	0.59b	0.54	0.59b	0.32	0.46b
Perennial	2.12	4.64a*	2.05	3.93a	2.06	5.14a	0.92	2.07a
Tissue								
Herbaceous	1.06	1b*	0.52	0.66b	0.42	0.41b	0.34	0.55b
Woody	2.88	6a*	3.8	5.28a	3.94	7.03a	1.68	2.94a
Seed category								
Dicot	2.24	5.04a	1.93	3.89b	1.47	4.04b	0.89	1.72b
Gymnosperm	1.92	3.74a	5.57	4.76a	8.63	9.49a	2.56	5.35a
Monocot	1.08	0.93b	0.63	1.35c	0.52	0.51c	0.35	0.79c
Leaf longevity								
Deciduous	2.96	5.5a	5.67	6.27a	4.48	5.71a	2.6	3.06a
Evergreen	3.1	6.95a	3.06	4.59b	4.74	8.64a	1.41	3.28b
Leaf form								
Broadleaf	3.59	7.43a	4.71	6.01b	4.12	7.12b	2.08	2.86a
Needle‐leaf	1.87	3.67b	5.02	5.21b	7.74	9.05a	2.29	4.93a
Photosynthetic pathway								
C_3_	2.04	4.69a	1.86	3.82a	2.04	5.14a	0.87	2.04a
C_3_–C_4_	1.13	0.63ab	0.85	0.69a	0.36	0.2b	0.37	0.24a
C_4_	1.75	1.85ab	1.37	2.65a	0.74	0.73b	0.71	1.29a
CAM	0.61	0.71b	2.57	2.43a	0.64	0.63b	0.76	0.52a

CAM, Crassulacean acid metabolism.

* These are the only two categories where rooting extents relative to aboveground volume showed significant differences between groups, where both annual and herbaceous plants had *D*_R_/*V*_S_ values greater than perennial and woody plants, therefore differing from the pattern shown by *D*_R_. *D*_R_ and *L*_R_ relative to shoot volume (*V*_S_) did not differ across all other classifications. The lowercase letters represent significant differences between groups via Tukey’s honest significance difference tests.

### Shifts in plant shape across climate

Whereas our initial analysis focused on factors influencing single measures of plant vertical or horizontal size, we further sought to understand how the shapes or dimensions of plants shift along climatic/resource gradients. To do this, we calculated four new plant shape ratios: two that we call ‘dimensional aspect’ ratios (*Y* : *X*_shoot_ and *Y* : *X*_root_) and two ‘shoot : root’ ratios (*S* :* R_y_* and *S* :* R_x_*). We plotted the four indicator ratios (Eqns (Eqn 1), (Eqn 2), (Eqn 3), (Eqn 4)) against a global climate gradient of aridity (Fig. 7, see later). Nonlinear regressions were fit to the mean ratio values for each aridity class.

The two‐dimensional aspect ratios (*Y *:* X*_shoot_ and *Y *:* X*_root_) depict a plant’s dimensions shifting towards either lengthening or widening their maximum extents (Eqns 1, 2). A high *Y* : *X* ratio represents a relative narrowing of plant morphology, whereas a low ratio represents a widening. As water availability increases, we expect to see relatively shallow plant growth belowground and a narrowing aboveground because plants may not need to root deeply in search of water and shoot heights are less limited by plant water potential. We calculated the dimensional aspect ratios as follows:

(Eqn 1) $$Y:X_{\text{shoot}}=\frac{H_{S}}{W_{S}}$$

(Eqn 2) $$Y:X_{\text{root}}=\frac{D_{R}}{W_{R}}$$

(*H*_S_, height of the plant; *W*_S_, aboveground width of the plant (shoot diameter); *D*_R_, maximum rooting depth; *W*_R_: maximum width of the root system). When *W*_R_ was not reported but *L*_R_ was, we used 2 *× L*_R_ in Eqn 2.

The second pair of growth indicator ratios, the shoot : root size ratios (*S* : *R_y_* and *S* : *R_x_*), depict a plant’s vertical and horizontal allometry (Eqns 3, 4). These metrics are similar to traditional shoot‐to‐root biomass ratios, but with biomass replaced by vertical length (*S* : *R_y_*) and horizontal width (*S* : *R_x_*). A high *S* : *R* ratio represents relatively greater aboveground investment, whereas a lower ratio represents relative belowground investment. We calculated the shoot : root size ratios as follows:

(Eqn 3) $$S:R_{y}=\frac{H_{S}}{D_{R}}$$

(Eqn 4) $$S:R_{x}=\frac{W_{S}}{W_{R}}$$

### Comparing individual plant rooting depth observations with ecosystem and plant‐functional‐type estimates

Because many terrestrial biosphere models rely on ecosystem‐level estimates of maximum rooting depth (Warren *et al*., 2015; McCormack *et al*., 2017; Drewniak, 2019), we compared how our rooting depth estimates for individual plants differ from ecosystem‐level estimates across biomes and climates. For ecosystem‐level data, we used the Root Profiles for Global Ecosystems (RPGE) dataset (Schenk & Jackson, 2002b) available online through the Oak Ridge National Laboratory Distributed Active Archive Center (Schenk & Jackson, 2003). We compared average individual plant rooting depth estimates by biome from the RSIP with (1) the ecosystem rooting depths (*D*_50_ and *D*_95_) by biome from the RPGE, and (2) the PFT rooting depth estimates used by the Energy Exascale Earth System Land Model (ELM; Fig. S2; Drewniak, 2019). ELM uses RPGE data to inform PFT rooting depth estimates (Drewniak, 2019).

To analyze the effect that climate parameters have on individual‐plant (*D*_R_) and ecosystem‐level rooting depths (*D*_50_ and *D*_95_), we used linear mixed effect regression models (LMERs) with biome as a random effect, the climate metrics as fixed effects, and rooting depth (*D*_50_, *D*_95_, and *D*_R_) as the dependent variable. The LMERs were performed using the lme4 package (Bates *et al*., 2015). We evaluated the LMERs using likelihood ratio tests, which compare the ANOVA of the full LMER with the fixed effects with the ANOVA of a null LMER with only random effects. Through the likelihood ratio test we computed the corrected Akaike information criterion AIC_c_ and *P*‐values to analyze only significant predictors (Winter, 2013; Hajduk & Bailey, 2017; Mazerolle, 2020). Using the model results for *D*_50_ and *D*_95_, we compared the standardized coefficients with that of individual plant maximum rooting depth *D*_R_ (Fig. S3).

## Results

### Rooting extents covary with shoot size

The two main plant rooting extents we examined, *D*_R_ and *L*_R_, differed substantially across growth forms, with woody plants, especially trees (mean *D*_R_ of 3.64 m), rooting the deepest and the widest (Fig. 4; Table 2). Semi‐shrubs, succulents, forbs, and grasses all had shallower, significantly indistinguishable rooting depths, with *D*_R_ being only *c*. 30% as deep as trees on average (Fig. 4a; Table 2; *P* < 0.001). Trees and shrubs had the widest lateral spreads (average *L*_R_ of 7.04 m and 3.33 m, respectively), whereas the average *L*_R_ for succulents was 2.22 m, 4.5 times wider than *L*_R_ for herbs (forbs and grasses; Fig. 4b; Table 2; *P* < 0.001). Relative to aboveground volume, *L*_R_ (*L*_R_/*V*_S_; Kruskal–Wallis *P* = 0.173) and *D*_R_ (*D*_R_/*V*_S_; Kruskal–Wallis *P* = 0.053) ratios did not significantly differ across growth forms.

**Fig. 5:**
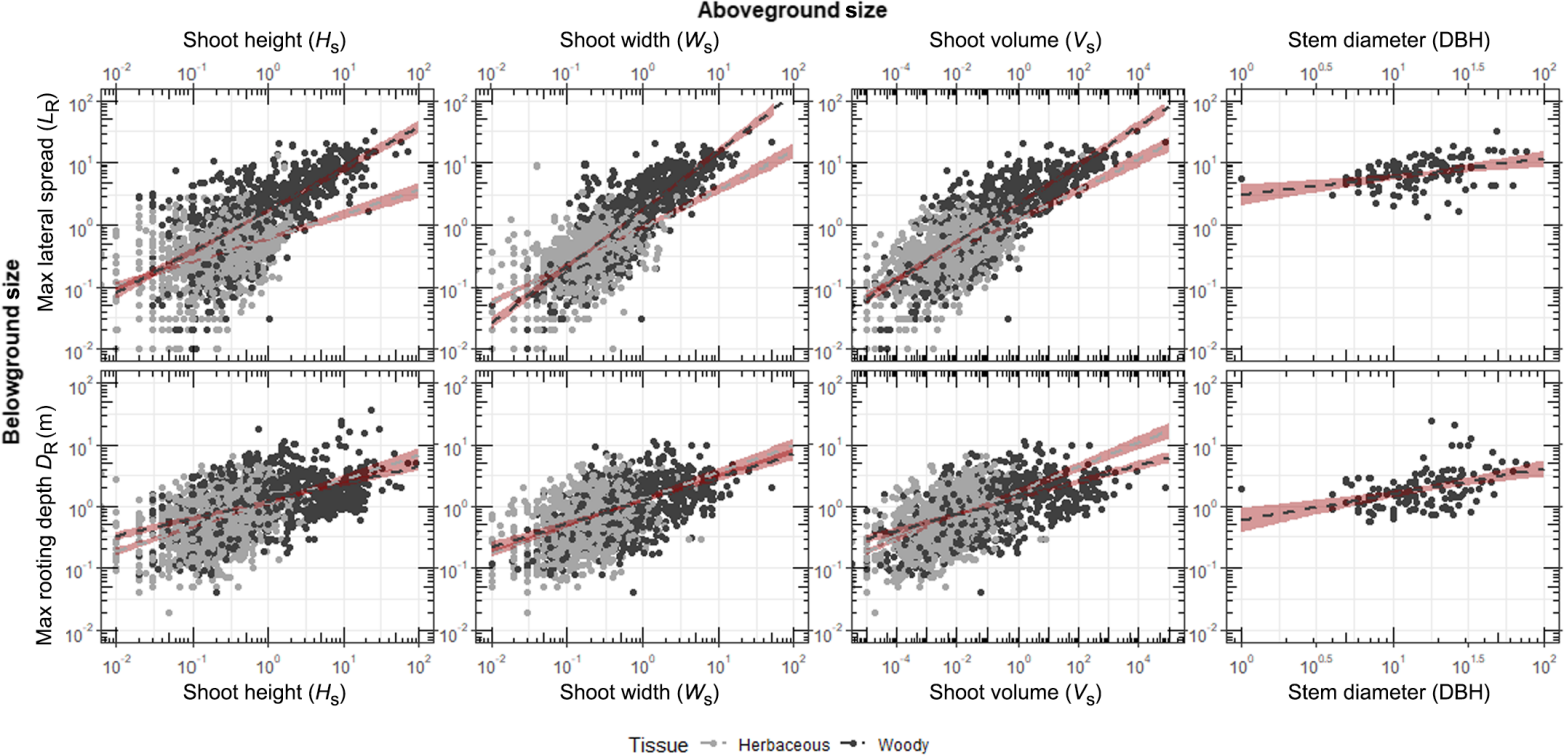
Scatter plots of plant root extents (*L*_R_, upper; *D*_R_, lower) against aboveground plant extents (*H*_S_, shoot height; *W*_S_, shoot width; *V*_S_, shoot volume; DBH, stem Diameter), with woody plants in dark gray and herbaceous plants in light gray. Shoot volume is calculated using the equation $V_{S}\;\left(m^{3}\right)=\pi \times H_{S}\times W_{S}^{2}/6$. The dashed lines (woody in dark gray and herbs in light gray) represent a linear regression where *P* < 0.05 in the form of *y = β + α* × *x*, and the red shaded regions are the 95% confidence interval. The statistics and the parameters for the linear regressions are in Table 3. The axes scales are in common log_10_.

Both rooting depth and spread scaled linearly with shoot size – specifically shoot height and width – across all growth forms (Fig. 5; Table 3). Whereas stem diameter (DBH) had a strong positive linear relationship with both maximum rooting depth and spread for trees (Table 3; *P* < 0.0001), *W*_S_ and *H*_S_ had stronger positive linear relationships with the rooting extents for both woody and herbaceous plants (Table 3).

**Table 3:** Linear and phylogenetically independent contrast (PIC) regressions of belowground extents (*D*_R_ and *L*_R_) to aboveground extents (*H*_S_, *W*_S_, *V*_S_, and DBH) in the form of *y* = *β*_0_ + *β*_1_ *x*, where *y* is *D*_R_ or *L*_R_, *β*_0_ is the intercept (Int.) and *β*_1_ is the slope.

	Max. rooting depth *D*_R_					Max. lateral spread *L*_R_				
	Linear regression			PIC		Linear regression			PIC	
	Int.	Slope (SE)	*R* ^2^ and *P*	Slope (SE)	*R* ^2^ and *P*	Int.	Slope (SE)	*R* ^2^ and *P*	Slope (SE)	*R* ^2^ and *P*
Shoot height *H*_S_										
All observations	0.04	0.34 (0.011)	0.29***	−0.19 (0.029)	0.028***	0.07	0.68 (0.016)	0.45***	−0.050 (0.035)	0.001
Woody	0.08	0.28 (0.016)	0.24***	0.19 (0.024)	0.118***	0.24	0.67 (0.024)	0.5***	0.40 (0.020)	0.504***
Herbaceous	0.05	0.39 (0.021)	0.2***	0.13 (0.045)	0.008*	−0.2	0.39 (0.025)	0.17***	0.51 (0.040)	0.155***
Shoot width *W*_S_										
All observations	0.09	0.39 (0.014)	0.28***	0.65 (0.020)	0.454***	0.15	0.81 (0.015)	0.6***	0.87 (0.019)	0.627***
Woody	0.09	0.38 (0.023)	0.27***	0.28 (0.038)	0.127***	0.26	0.93 (0.024)	0.68***	0.57 (0.031)	0.449***
Herbaceous	0.09	0.4 (0.022)	0.2***	0.41 (0.030)	0.173***	−0.1	0.56 (0.022)	0.34***	0.47 (0.027)	0.251***
Shoot volume *V*_S_										
All observations	0.15	0.16 (0.005)	0.33***	0.23 (0.010)	0.275***	0.27	0.3 (0.0053)	0.63***	0.33 (0.010)	0.459***
Woody	0.13	0.13 (0.0077)	0.29***	0.083 (0.012)	0.122***	0.35	0.31 (0.0084)	0.67***	0.18 (0.009)	0.527***
Herbaceous	0.23	0.19 (0.0084)	0.29***	0.17 (0.013)	0.161***	0.08	0.24 (0.0084)	0.4***	0.23 (0.012)	0.311***
Stem diameter DBH										
Trees	1.09	0.41 (0.08)	0.16**	0.50 (0.10)	0.221***	0.76	0.51 (0.11)	0.16**	0.31 (0.10)	0.112*

***, *P* < 0.0001; **, *P* < 0.001; *, *P* < 0.01. PIC regression intercepts (*β*_0_) set to zero.

### High phylogenetic signals for woody plant root system lateral spreads (*L*_R_) and aboveground size (*H*_S_, *W*_S_, *V*_S_, and DBH)

Pagel’s lambda values for woody plants showed high phylogenetic signals for aboveground size traits (*H*_S_ *λ* = 0.934; *W*_S_ *λ* = 0.750; *V*_S_ *λ* = 1.0; DBH *λ* = 0.922) and for root lateral spread (*L*_R_ *λ* = 0.865; Table S3). Lambda values for herbaceous plants were much lower than those of woody plants, suggesting a lower phylogenetic signal, except for maximum rooting depth, where the phylogeny of herbs accounted for more of the variation in *D*_R_ values (herb *D*_R_ *λ* = 0.644 and woody *D*_R_ *λ* = 0.271; Table S3). For herbaceous *L*_R_, *W*_S_, and *V*_S_ the phylogeny accounted for little to no variation in trait values across species (*λ* < 0.2), whereas phylogeny had a moderate effect on shoot height (*H*_S_ *λ* = 0.558).

There remained a positive relationship between shoot size and root system size (Table 3), even when using phylogenetically independent contrasts for woody and herbaceous plants, except for the relationship between *H*_S_ and belowground extents (*D*_R_ and *L*_R_) when combining woody and herbaceous plants (Figs S4a, S5a). The negative PIC slopes (*β*_1_) for *D*_R_ (*β*_1_ = −0.19, *P* < 0.0001) and *L*_R_ (*β*_1_ = −0.05, *P* = 0.18) when regressed against *H*_S_ were due to the strong phylogenetic signal for *H*_S_ (*λ* = 0.985), and the large differences between the shoot heights of woody and herbaceous plants (Table 3; Figs S4a, S5a). Overall, the PIC regressions and correlations of above to belowground plant size (Figs S4, S5) tended to be consistent with the linear relationships between plant extents (Fig. 5; Table 3).

### Plant size extents across morphological and leaf traits

Plant size extents differed significantly across the leaf and morphological traits we collected (i.e. lifespan, tissue, seed category, leaf longevity, leaf form, and photosynthetic pathway; Table 2). The average absolute extents (*D*_R_ and *L*_R_) of perennials were more than six times greater than for annuals, but their extents relative to shoot volume were four times greater (Table 2). The *D*_R_ and *L_R_* of woody plants were, respectively, six times and 10 times greater than forherbs, but the *D*_R_/*V*_S_ of herbs was 2.3 times greater than for woody plants.

Among woody plants (trees and shrubs), deciduous plants had lateral spreads that were an average of 5.67 m (twice the width of evergreens), and broadleaf plants had an average *D*_R_ of 3.59 m (two times deeper than needle‐leaf plants) (Table 2). Aboveground, we found similar trends, with perennial and woody plants having greater shoot heights *H*_S_ and widths *W*_S_ (Table 2) than annual and herbaceous plants did. Deciduous trees had average shoot widths of 2.6 m, which is twice that of evergreen trees. The *H*_S_ values of needle‐leaf plants were 7.74 m, also two times the *H*_S_ of broadleaf plants, whereas *D*_R_ was two times deeper for broadleaf plants than for needle‐leaf plants.

### Deeper roots in drier and more seasonal climates

We found significant linear relationships between the rooting extents and the primary climate metrics (MAE, MAP, *A*_i_, and *S*_a_; see Table 1 for abbreviation definitions) we analyzed (Fig. S6; Table S4). Rooting depth *D*_R_ correlated positively with MAE and negatively with *A*_i_ and MAP (*P* < 0.0001). Lateral spread *L*_R_ was positively related to MAP and *A*_i_ for all plants. *L*_R_ was negatively related to MAE for herbs, and with *S*_a_ for woody plants (*P* < 0.0001; Fig. S6; Table S4).

Though the PIC results tended to agree with the trends shown with the linear regressions (Table S4; Figs S6–S8) there were a few instances where the trends of the PIC results differed from the log‐linear regressions. For example, there was a positive linear relationship between *L*_R_ and MAP for woody plants (*β*_1_ = 0.21 ± 0.04; *P* < 0.0001; Table S4), whereas the PIC regression showed a negative relationship (*β*_1_ = −0.45; *P* < 0.0001; Table S4).

### Differences in predictor importance for shoot and root extents

**Fig. 6:**
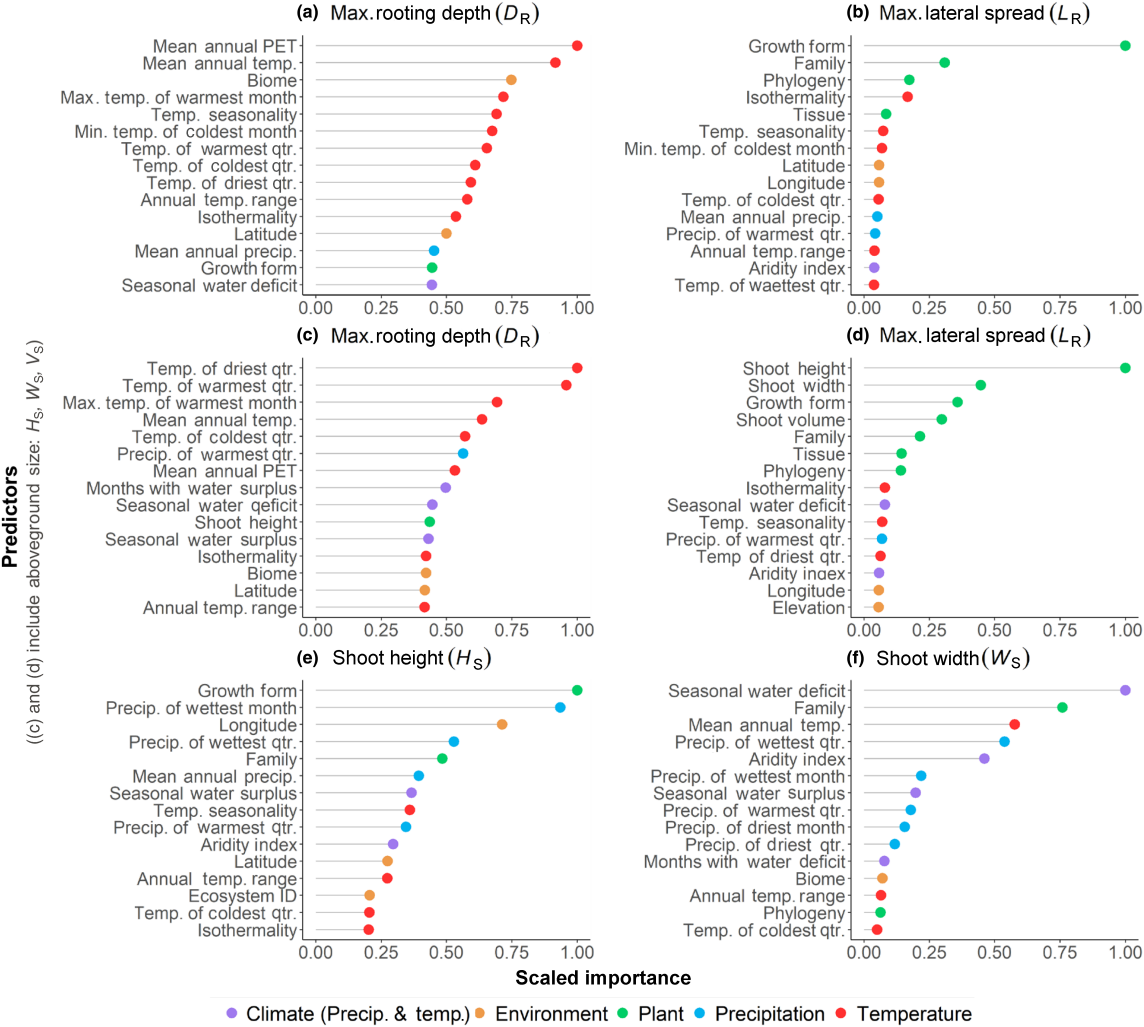
Random forest variable importance for (a) maximum rooting depth *D*_R_, (b) maximum lateral spread *L*_R_, (c) *D*_R_ with shoot size included as predictors, (d) *L*_R_ with shoot size as predictors, (e) shoot height *H*_S_, and (f) shoot width *W*_S_. The predictors are colored by predictor type, according to the figure key. The *y*‐axis is the 15 most important predictors in descending order, and the *x*‐axis is a scaled variable importance. Scaled variable importance = variable importance[*i*] ÷ max(variable importance). The predictors used for the random forest analysis can be found in the fourth column of Supporting Information Table S1. Measures of aboveground size were not included as predictors in (a) and (b), whereas shoot height, shoot width, and shoot volume *V*_S_ were included as predictors in (c, d). Belowground extents (*D*_R_ and *L*_R_) were not included as predictors for *H*_S_ and *W*_S_. [Correction added after first publication 8 March 2022: panel (c) in Fig. 6 has been corrected.]

Our random forest approach highlighted the important predictors for each of the plant size extents, with climate and temperature seasonality being important for *D*_R_ and shoot size and plant characteristics being the most important for *L*_R_ (Fig. 6a–d). Climate descriptors such as MAE, mean annual temperature, temperature seasonality, and maximum temperature were the most important predictors of *D*_R_ (Fig. 6a,c), with *D*_R_ increasing with warmer and more seasonal climates. *L*_R_ was mostly affected by shoot size (*H*_S_ and *W*_S_, Fig. 6d) and plant descriptors (i.e. growth form and family; Fig. 6b,d). Partial dependencies showed that *L*_R_ was greatest in woody plants, and in less seasonal climates (i.e. climates where temperature seasonality < 500, annual temperature range < 25°C, and isothermality > 50). When shoot size (*W*_S_, *H*_S_, and *V*_S_) was omitted from the random forest analyses (Fig. 6a,b) it had little effect on the variable importance ranking for *D*_R_, but it led to growth form, family, phylogeny, and isothermality becoming the most important variables for predicting *L*_R_.

Aboveground, the growth form and family were among the most important predictors of *H*_S_ and *W*_S_, followed by various climate metrics (Fig. 6c,d). The partial dependencies showed that trees, and plant families primarily made up of trees, represented the greatest *H*_S_ and *W*_S_ values. Additionally, *H*_S_ and *W*_S_ were greatest in less arid climates (*A*_i_ > 1). *H*_S_ was greatest in climates with high precipitation and high seasonal water surplus (*P*_sur_ > 0.3). *W*_S_ was greatest in climates with low seasonal water deficits (*P*_def_ < 0.2) and colder climates (mean annual temperature < 10°C).

### Divergence in woody plant dimensions across aridity

**Fig. 7:**
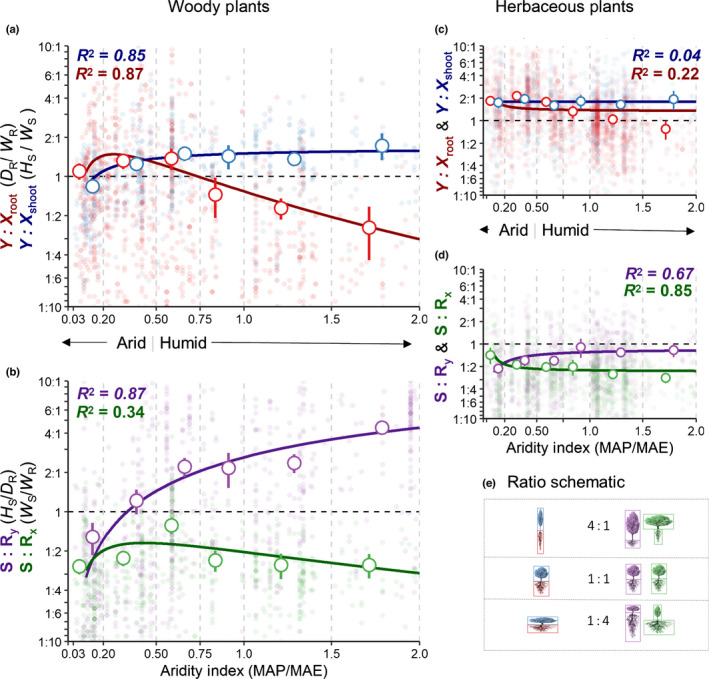
Woody plant shapes diverge across aridity classes. Point range represents the mean and confidence intervals grouped by aridity index categories for the four shape ratios with fitted nonlinear regressions. (a) Dimensional aspect ratios for woody plants, (b) shoot : root ratios for woody plants, (c) dimensional aspect ratios for herbaceous plants, and (d) shoot : root ratios for herbaceous plants. Either log‐normal ($y=a\exp\{‐0.5{\left[\log_{e}\left(x/x_{0}\right)/b\right]}^{2}\}$) or nonlinear saturation curves (y=ax/(b+x)) were fitted to the means of each shape ratio across aridity classes, and the *R* ^2^ values are stated (see Supporting Information Table S5 for full equations). The aridity index categories, delimited by light gray dashed lines, are arid (0–0.2), semi‐arid (0.2–0.5), subhumid (0.5–0.75), humid (0.75–1.0), per‐humid (1.0–1.5), and hyper‐humid (1.5–2.0). The dashed black line represents ratio values equal to one. (e) The color‐coded schematics represent the relative dimensions for each ratio value. MAP, mean annual precipitation; MAE, mean annual potential evapotranspiration; *D*_R,_ max rooting depth; *W*_R_, root system width, *H*_S_, shoot height; *W*_S_, shoot width.

The dimensions of woody plants shifted towards deeper and narrower root systems in more arid climates and towards taller and narrower shoots in relatively humid climates (Fig. 7a). Significant shifts in *Y* : *X* and *S* : *R* values with climate were seen only for woody plants (Fig. 7; Table S5). The aspect ratios of shoots and roots (*Y* : *X*_shoot_ and *Y* : *X*_root_) for woody plants in arid climates (*A*_i_ < 0.2) did not differ significantly (*P* = 0.308) (Fig. 7a). The average *Y* : *X*_shoot_ and *Y* : *X*_root_ values of woody plants in climates where *A*_i_ < 0.5 were 1.8 and 1.3, respectively. As *A*_i_ increased, the aspect ratio curves diverged, crossing at an *A*_i_ of 0.43, near the arid–humid threshold (*A*_i_ = 0.5; Fig. 7a). The *Y* : *X*_shoot_ curve saturated in humid climates (*A*_i_ > 0.5). *Y* : *X*_root_ decreases as climates become more humid, with root systems being wider relative to their depth (*Y* : *X*_root_ < 1) at an *A*_i_ of 0.73. The average *Y* : *X*_shoot_ and *Y* : *X*_root_ values of woody plants in humid climates were 1.6 and 0.7, respectively.

Woody plants, on average, had shoots taller relative to rooting depth in humid climates (*S* : *R_y_* > 1 when *A*_i_ > 0.34), but in arid climates the rooting depth is generally greater than shoot height (Fig. 7b). In arid climates, woody plants tended to be both wider and deeper belowground than aboveground, with *S* : *R* values < 1 (Fig. 7b, purple). Horizontal allometry does not shift much across *A*_i_; and the mean *S* : *R_x_* is 0.44, indicating that woody plants are, on average, more than two times wider belowground than aboveground. Herb *S* : *R* and *Y* : *X* values do not shift substantially across *A*_i_ (Fig. 7c,d), with mean *S* : *R* values < 1 (*S* : *R_y_* = 0.654; *S* : *R_x_* = 0.452), indicating that herbs generally take up more vertical and horizontal space belowground relative to aboveground dimensions.

### Comparing RSIP individual plant data to broader scale estimates of maximum rooting depth

**Fig. 8:**
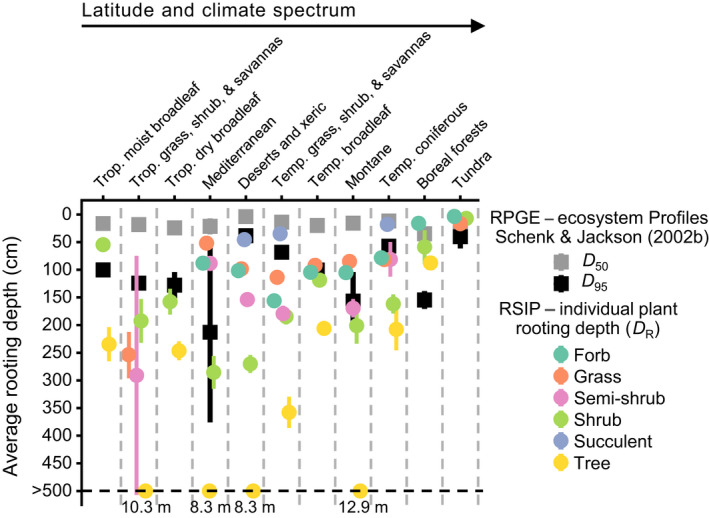
Average maximum rooting depths per biome (±SE) for the Root Profiles for Global Ecosystems (RPGE) in black and gray squares. The Root Systems of Individual Plants (RSIP) maximum rooting depths *D*_R_ are averaged by biome growth form and biome (colored circles). When the average rooting depth exceeds 5 m, that depth is explicitly stated. *D*_50_ (gray squares) is the 50^th^ percentile rooting depth for ecosystem profiles, and *D*_95_ (black squares) is the effective maximum rooting depth for an ecosystem, the 95^th^ percentile rooting depth (Schenk & Jackson, 2002b).

We compared the average RSIP maximum rooting depths (*D*_R_) across growth forms and biomes with the biome‐based estimates from the RPGE (*D*_95_; Fig. 8). For all biomes with trees, the average tree *D*_R_ was significantly deeper than *D*_95_ (ecosystem‐scale maximum rooting depth; Table 1), sometimes by several meters, except for boreal forests, where *D*_95_ was deeper. Tropical and seasonally dry climates had the largest disagreement between the RPGE and RSIP values, with *D*_R_ values for multiple growth forms being significantly deeper than *D*_95_ (Fig. 8). The ELM PFT parameters closely resembled RPGE *D*_95_ estimates, except that ELM assigns tropical forest trees a maximum rooting depth of 3 m (Drewniak, 2019).

The ecosystem‐scale *D*_50_ (50^th^ percentile rooting depth) was more sensitive to climate than the individual‐plant (*D*_R_) and ecosystem‐scale (*D*_95_) rooting depths were. The *D*_50_ climate coefficients are greater than the *D*_R_ coefficients, showing that *D*_50_ is more heavily skewed by climate variables than *D*_R_ is (Fig. S3). This is exemplified by the slope of the linear regression across the coefficients, where a unit slope is a one‐to‐one relationship between *D*_R_ and *D*_50_ coefficients; however, the slope was 0.52 with an *R* ^2^ = 0.81 (*y* = 0.52*x* − 0.016; Fig. S3). The opposite was true for *D*_95_ vs *D*_R_, where the slope of the regression across the climate coefficients was 2.62 (*y* = 2.62*x* − 0.04, *R* ^2^ = 0.71; Fig. S3), indicating that individual plant rooting depth *D*_R_ is more variable across climates than ecosystem‐level rooting depth *D*_95_ is. At both the individual plant and ecosystem scales, roots were deeper in climates with higher mean and maximum temperatures and in climates with greater seasonal deficits of precipitation (Fig. S3). Shallower roots were found in humid climates and in climates with greater surpluses of precipitation (Fig. S3).

## Discussion

Using our expanded RSIP database, we found the following patterns in plant size and shape globally: shoot size and root system size strongly covary; water availability and plant characteristics greatly influence shoot size, whereas rooting depth is primarily influenced by temperature seasonality and lateral spread by shoot size; woody plants have deeper, narrower root systems in more arid climates and taller shoots in humid climates; and estimates of the depth and lateral spread of plant root systems are likely underestimated at the global scale.

### Shoot size covaries strongly with root system size across plant functional groups

Both rooting depth *D*_R_ and lateral spread *L*_R_ scale linearly with aboveground size extents (*H*_S_, *W*_S_, *V*_S_, and DBH; Table 3), as expected with allometric allocation (Niklas & Enquist, 2001; Enquist & Niklas, 2002). Furthermore, multiple tropical forest studies have highlighted the link between shoot size and root system size (Ivanov *et al*., 2012; Brum *et al*., 2019; Smith *et al*., 2019). Of all the aboveground variables, shoot width *W*_S_ had the strongest positive relationship to rooting depth and spread across all plants (Table 2). Traditionally, stem diameter DBH is the most common metric used for allometric scaling in forestry and plant physiology (Cermák *et al*., 1998; Ledo *et al*., 2017), including long‐standing allometric relationships between DBH and crown radius (Dawkins, 1963; O’Brien *et al*., 1995). DBH has also been used to estimate coarse root biomass (Tobin *et al*., 2007; Gou *et al*., 2017) and effective rooting depth (Brum *et al*., 2019). However, though DBH strongly correlated with shoot height and width, DBH did not correlate with rooting depth in our analysis (Fig. S9). We suggest that shoot width may be better used to estimate the size of root systems, as it correlates positively with both rooting depth and spread (Table 3; Fig. S9).

As expected, woody plants, especially trees, had the largest rooting (*D*_R_ and *L*_R_) and shoot extents (*W*_S_, and *H*_S_) and herbs had the smallest extents. Succulents have unique root system shapes, having the shallowest root systems yet wider lateral extents than herbs do (Fig. 4). One explanation for the shape of succulent root systems may be that succulents are found in regions with extremely dry soils, where shallow and elongated root systems are adapted to acquiring intermittent rainfall and fog (Jordan & Nobel, 1984; February *et al*., 2013). Additionally, there can be a large degree of rooting depth plasticity within the same species and environment. For example, a study on the rooting depth of *Panicum maximum*, a tropical perennial bunchgrass, in the state of São Paulo, Brazil, found rooting depths for mature grasses ranging from 0.85 to 4.85 ms across > 50 observations (Villares *et al*., 1953).

We found that the rooting depths of annuals and herbs relative to shoot volume (*D*_R_/*V*_S_) were much greater than that of perennials and woody plants, demonstrating an investment in belowground organs by shorter‐lived plants. John Ernest Weaver (1958) observed the rooting patterns of forbs and found that some forbs can root deeply below the root zone of neighboring plants to avoid competition, quickly occupying depths of greater than 1.2 m in their first growing season and up to 4.6 m at maturity. The deepest‐rooted forb in the RSIP, *Alhagi maurorum* – commonly known as camelthorn – reached rooting depths of 20 m but rarely exceeded 1.2 m in height (Nechaeva, 1985). The need of some plants to root deeply could also be due to competition in the form of root territoriality or resource depletion (Schenk, 2006, 2008a). The ability of plants such as herbs (which we often think of as being ‘small’ aboveground) to root at times several meters in depth is surprising.

Maximum lateral root spreads were strongly influenced by shoot size, even more than maximum rooting depth (Figs 5, S9; Table 3), further evidenced by the high variable importance of shoot height, width, and volume in the lateral extent random forest model (Fig. 6d). Modeling studies have demonstrated that lateral roots are more efficient at anchoring larger aboveground plants than deep roots are (Ennos, 1993), and a strong linear relationship has been found between lateral spread and stem diameter (Schwarz *et al*., 2010). The relationship between lateral extent and shoot size highlights the potential importance of lateral root reinforcement for shoot anchorage.

Not surprisingly, there were strong phylogenetic signals for several size and shape traits, especially for aboveground traits, but also for the lateral spread of root systems. Plant species within a genus tend to be similar in growth form, and many plant families consist predominantly of woody plants, herbs, or succulents; and some woody families include mostly trees, whereas others include mostly shrubs and smaller trees. Different environments that favor trees, smaller woody plants, or herbs will therefore cause ecological and evolutionary sorting of genera and families, and historical effects will contribute to this sorting as well (Herrera, 1992), leaving what appears to be a strong phylogenetic signal in plants sizes and shapes (see Table S3).

### The effect of temperature and precipitation seasonality on rooting depth

Relationships among above and belowground plant traits that we found are not static across the climate space. Our results agree with Schenk & Jackson (2002a), who found that plants root deeper relative to shoot size in arid climates. A global meta‐analysis of forest biomass allocation found contrasting results, where root biomass decreased with temperature – analogous with MAE – but found no relation with aridity (Reich *et al*., 2014), potentially highlighting the difference between the space occupied by roots and overall biomass. A decrease in root system size relative to shoot size as climates become less arid would be expected under a plant resource economics framework, where increased water availability would allow plants to invest in aboveground growth when they are no longer limited by water availability belowground (Shipley & Meziane, 2002; Farrior *et al*., 2015; Anderegg *et al*., 2016; O'Brien *et al*., 2017).

Metrics of climate seasonality, specifically temperature seasonality and proxies for water availability, are important for understanding global rooting patterns. The relationship between precipitation seasonality and deep rooting has been well documented in seasonally dry ecosystems (Nepstad *et al*., 1994; Oliveira *et al*., 2005; Singh *et al*., 2020), although predicting deep rooting using global climate metrics is difficult given the complexities of plant–soil–water interactions. However, we provide ample evidence that deeper roots are more likely to occur in arid climates with hotter temperatures and seasonal precipitation (Figs 6a, S3, S6).

The exact relationships between rooting extents and seasonality are still unclear because we need root data at finer scales coupled with measures of seasonality that serve as better proxies for plant‐available water, such as plant‐accessible water storage capacity, dry‐season water drawdown, and climatic water deficit (Fellows & Goulden, 2017; Ledo *et al*., 2017; Klos *et al*., 2018). Additionally, as climates change, metrics of interannual seasonality may provide insight on the climates that a plant is adapted to and its rooting response (Fischer *et al*., 2013; Pratt & Mooney, 2013; Stocker *et al*., 2013; Knapp *et al*., 2015). For instance, a study in an arid grassland found that increased interannual variability in precipitation causes a shift in community composition towards deeply rooted shrubs (Weltzin & McPherson, 2000; Gherardi & Sala, 2015). One promising method to understand spatial patterns in maximum rooting depth is to consider the climatology of the cumulative water deficit to estimate the rooting zone water storage capacity to which plants are adapted (Gao *et al*., 2014; Stocker *et al*., 2021).

### Above and belowground woody plant geometries diverge across climates

Woody plants shift their shapes across climates more than other plant types do (i.e. herbs), with woody root systems being relatively narrower in arid climates and relatively wider in humid climates (Fig. 7a, red). In arid climates, woody plants are short and wide aboveground (*Y* : *X*_shoot_ < 1); an important transition occurs at the semi‐arid to subhumid boundary, where plants become taller in relation to their width (Fig. 7a). Shoot height increases as plants compete for light, especially when plants are no longer limited by other resources (Falster & Westoby, 2003; Craine & Dybzinski, 2013). In humid climates, the aspect ratio of shoots (Fig. 7a, blue) does not change much, potentially because of the biological limits to the possible shoot size that plants can support and plants could be limited by other resources (Reich *et al*., 2003; Koch *et al*., 2004; Westoby & Wright, 2006; Niklas, 2007; Moles *et al*., 2009; Krishnamurthy, 2015). Overall, the shapes of woody plants above and belowground diverge across the climate space, where, as aridity decreases, root systems widen and shoots narrow (Fig. 7a). The aspect ratio of root systems decreases with increasing humidity, representing a relative widening of root systems. This could demonstrate a shift in resource priority, where, as plants become less limited by water availability, root systems may prioritize lateral growth to increase nutrient foraging (Lynch, 2005) and to anchor larger aboveground plants (Gilman, 1990; Dupuy *et al*., 2007).

Woody and herbaceous plants’ root systems exhibit widths that are more than twice their shoot widths on average (*S* : *R_x_* values of 0.44 and 0.45, respectively). Our results are consistent with the literature review by Schwarz *et al*. (2010), who found that the lateral radius of tree roots is typically one to three times the shoot radius. The greater widths reached by plants belowground contradicts the common misconception that the width of root systems mirrors the width of shoots (Day *et al*., 2010; Sinacore *et al*., 2017). For example, a whole‐tree harvest study found that, unlike the tightly packed crowns of forest trees, roots overlap greatly with their neighbors, resulting in root system radii being twice that of crown radii (Sinacore *et al*., 2017). We postulate that *S* : *R_x_* may display plasticity across other resource and competition gradients, such as nutrients belowground (Lynch, 2005), light aboveground (Takenaka, 1994; Cermák *et al*., 1998; Vieilledent *et al*., 2010), or increased competition with neighboring plants (Schenk *et al*., 1999; Schenk, 2006; Cahill *et al*., 2010; Lepik *et al*., 2021).

Whereas woody plants growing in more arid climates had deeper, narrower root systems than woody plants in humid climates did, herbs – forbs and grasses – did not show the same trend. Herb root systems may rely on other trait‐based strategies to cope with resource stress (Roumet *et al*., 2016; Freschet *et al*., 2018; Wang *et al*., 2020), such as going dormant or shedding fine roots during the dry season (Eissenstat & Yanai, 1997), increasing root density to avoid dehydration (Norton *et al*., 2016; Singh *et al*., 2020), and optimizing for fast resource uptake by having a high specific root length (Roumet *et al*., 2006). However, we did find that herbs occupy much more space belowground compared with aboveground (Fig. 7), which could be part of a stress or disturbance‐coping strategy (Singh *et al*., 2020).

### Are we underestimating plant rooting depth?

The RPGE dataset has been a primary source of rooting depth data used by many Earth system models, usually incorporated to parameterize biome‐level or PFT rooting depth distributions (Schenk & Jackson, 2002b, 2005; Warren *et al*., 2015). For example, the US Department of Energy's ELM uses the RPGE to inform its PFT maximum rooting depth, a static parameter, with the exception that tropical tree PFT maximum rooting depths were set to 3 m based on expert opinion (Fig. S2; Drewniak, 2019), considerably deeper than the RPGE estimates. Across several biomes, our analysis found that RSIP rooting depths averaged by growth form were much deeper than the RPGE biome‐level estimates (Fig. 8). This is especially true for biomes with high seasonality and deeply rooted woody plants, such as tropical, Mediterranean, xeric, and forested biomes (Fig. 8).

Comparing RSIP *D*_R_ values with the RPGE, we found that *D*_50_ was very sensitive to changes in temperature, whereas individual plant maximum rooting depth *D*_R_ was slightly more sensitive to climate parameters than *D*_95_ was (Fig. S3). The correlations between climate and estimates of rooting depths are important because the estimates are generally used to characterize entire biomes or PFTs without considering environmental changes within biomes. Furthermore, studies have shown that terrestrial‐biosphere models are sensitive to changes in plant rooting depth, leading to significant global variations in gross primary productivity, evapotranspiration, nitrogen uptake, and more – suggesting a more accurate and dynamic approach to modeling the size of plant root systems is needed (Kleidon & Heimann, 1998; Warren *et al*., 2015; McCormack *et al*., 2017; Drewniak, 2019). Based on our findings, we suggest the following: rooting depth distribution should be modelled dynamically, by accounting for resource availability and plant optimality, as suggested in previous studies (Schenk, 2008b; Drewniak, 2019), and that the RSIP *D*_R_ data could be used to parameterize maximum rooting depth across PFTs, whereas the RPGE *D*_50_ could inform the relative distribution of roots within the vertical soil column.

### Significance and pitfalls

Our study provides a global synthesis of maximum plant extents and dimensions and shows that the lateral spread of root systems covaries strongly with aboveground plant size, whereas rooting depth is much more influenced by temperature and climate seasonality. As suggested by Tumber‐Dávila & Malhotra (2020), in addition to climate variables, future studies should also focus on root system characteristics across resource gradients. Future studies could also characterize plant volumes above and belowground more explicitly. There are additional environmental constraints on root systems that should be investigated, such as the temporal or vertical availability of plant‐accessible water and plant–soil interactions that we were unable to accurately test at the global scale, leaving a need for additional studies at the ecosystem or individual plant scales (Brantley *et al*., 2017; Erktan *et al*., 2018).

We present novel findings on relationships of plant size and shape above and belowground, and across the climate spectrum. Given that aboveground plant size is a major axis of variation in the global spectrum of plant form and function (Díaz *et al*., 2016; Joswig *et al*., 2022) and that our results characterized strong links between above‐and belowground plant size, our analysis and the RSIP can contribute to an improved understanding of plant size trade‐offs above and belowground. Better predicting these trade‐offs would have far‐reaching consequences for understanding nutrient, water, and carbon cycling of ecosystems.

## Author contributions

SJT‐D wrote the manuscript with critical input and revisions from RBJ, HJS and ED. All authors contributed significantly to the design of the study and the analyses. HJS and RBJ created the original RSIP database that the study builds upon.

## Supporting information


**Dataset S1** Root systems of individual plants database.

**Fig. S1** Map of Root Systems of Individual Plants Database (RSIP) observations by versions.
**Fig. S2** Comparison of Root Profiles for Global Ecosystems (RPGE) rooting depth estimates to the plant functional type (PFT) estimates.
**Fig. S3** The effect that climate variables have on individual plant rooting depth vs ecosystem‐scale rooting depth.
**Fig. S4** PIC of maximum rooting depth (*D*_R_) to aboveground plant size (*H*_S_, *W*_S_, *V*_S_, and DBH).
**Fig. S5** PIC of maximum lateral spread (*L*_R_) to aboveground plant size (*H*_S_, *W*_S_, *V*_S_, and DBH).
**Fig. S6** The influence of climate metrics on max rooting depth (*D*_R_) and maximum lateral spread (*L*_R_).
**Fig. S7** PIC of maximum rooting depth (*D*_R_) to climate metrics (MAE, MAP, *A*_i_ and *S*_a_).
**Fig. S8** PIC of maximum lateral spread (*L*_R_) to climate metrics (MAE, MAP, *A*_i_ and *S*_a_).
**Fig. S9** Correlation matrix for the above and belowground plant size metrics.
**Table S1** Description of RSIP parameters (*n* is the total number of observations).
**Table S2** RSIP categorical groups. The number of total observations *n*, and unique species, geographic locations, and studies for each class are shown.
**Table S3** Pagel’s lambda values for the above and belowground plant measurements.
**Table S4** Comparison of absolute extents (*D*_R_ and *L*_R_) with climate metrics.
**Table S5** Nonlinear regression curves for the shape ratios plotted in Fig. 7.
Please note: Wiley Blackwell are not responsible for the content or functionality of any Supporting Information supplied by the authors. Any queries (other than missing material) should be directed to the *New Phytologist* Central Office.

## Data Availability

The RSIP can be found as Dataset S1. The RPGE data (Schenk & Jackson, 2003) are openly available in the ORNL DAAC at https://doi.org/10.3334/ORNLDAAC/660.
