# Supplementary material for: Plant sizes and shapes above and belowground and their interactions with climate
Source: New Phytol. 2022 Mar 8;235(3):1032–56. doi: 10.1111/nph.18031 (PMC9311740; doi:10.1111/nph.18031)
Supplement: Supplementary file 2 — Fig. S1 Map of Root Systems of Individual Plants Database (RSIP) observations by versions. Fig. S2 Comparison of Root Profiles for Global Ecosystems (RPGE) rooting depth estimates to the plant functional type (PFT) estimates. Fig. S3 The effect that climate variables have on individual plant rooting depth vs ecosystem‐scale rooting depth. Fig. S4 PIC of maximum rooting depth (D R) to aboveground plant size (H S, W S, V S, and DBH). Fig. S5 PIC of maximum lateral spread (L R) to aboveground plant size (H S, W S, V S, and DBH). Fig. S6 The influence of climate metrics on max rooting depth (D R) and maximum lateral spread (L R). Fig. S7 PIC of maximum rooting depth (D R) to climate metrics (MAE, MAP, A i and S a). Fig. S8 PIC of maximum lateral spread (L R) to climate metrics (MAE, MAP, A i and S a). Fig. S9 Correlation matrix for the above and belowground plant size metrics. Table S1 Description of RSIP parameters (n is the total number of observations). Table S2 RSIP categorical groups. The number of total observations n, and unique species, geographic locations, and studies for each class are shown. Table S3 Pagel’s lambda values for the above and belowground plant measurements. Table S4 Comparison of absolute extents (D R and L R) with climate metrics. Table S5 Nonlinear regression curves for the shape ratios plotted in Fig. 7. Please note: Wiley Blackwell are not responsible for the content or functionality of any Supporting Information supplied by the authors. Any queries (other than missing material) should be directed to the New Phytologist Central Office. [file NPH-235-1032-s002.pdf]

New Phytologist **Supporting Information**

Article title: Plant sizes and shapes above- and belowground and their interactions with climate
Authors: Shersingh Joseph Tumber-Dávila, H. Jochen Schenk, Enzai Du, and Robert B. Jackson
Article acceptance date: 30 January 2022

The following Supporting Information is available for this article:

Dataset S1 Root Systems of Individual Plants Database and manuscript tables. (Provided as a separate file)

Fig. S1 Map of RSIP observations by versions.

Fig. S2 Comparison of Root Profiles for Global Ecosystems rooting depth estimates to the plant functional type (PFT) estimates.

Fig. S3 The effect that climate variables have on individual-plant rooting depth versus ecosystem-scale rooting depth.

Fig. S4. PIC of max rooting depth (D_R) to aboveground plant size (H_S, W_S, V_S, & *DBH*).

Fig. S5. PIC of max lateral spread (L_R) to aboveground plant size (H_S, W_S, V_S, & *DBH*).

Fig. S6 The influence of climate metrics on max rooting depth (D_R) and max lateral spread (L_R).

Fig. S7 PIC of max rooting depth (D_R) to climate metrics (*MAE*, MAP, A_i, & S_a).

Fig. S8 PIC of max lateral spread (L_R) to climate metrics (*MAE*, MAP, A_i, & S_a).

Fig S9 Correlation matrix for the above- and belowground plant size metrics.

Table S1 Description of RSIP Parameters (n is the total number of observations).

Ta**ble S2** RSIP categorical groups.

Table S3 Pagel's Lambda values for the above- and belowground plant measurements.

Table S4 Comparison of absolute extents (D_R & L_R) to climate metrics.

Table S5 Nonlinear regression curves for the shape ratios plotted on Fig. **7**.

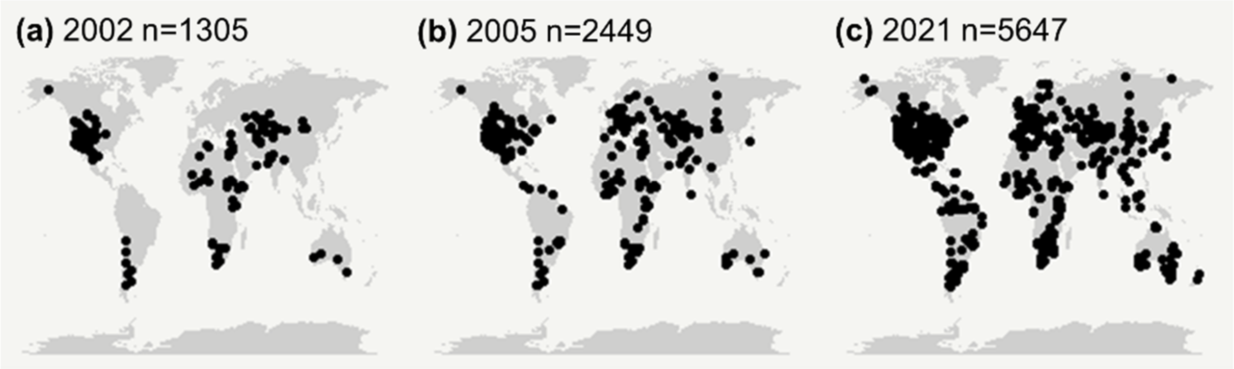

Fig. S1 Maps of Root Systems of Individual Plants (RSIP) observations by versions. (a) The original, first published, version of the RSIP database included 1305 entries for water-limited systems with ≤1,000 mm mean annual precipitation (Schenk & Jackson, 2002a). (b) Version 2, published in 2005, expanded the RSIP to 2449 observations spanning all climates (Schenk & Jackson, 2005). (c) Our new RSIP with 5,647 total observations, a more than two-fold expansion.

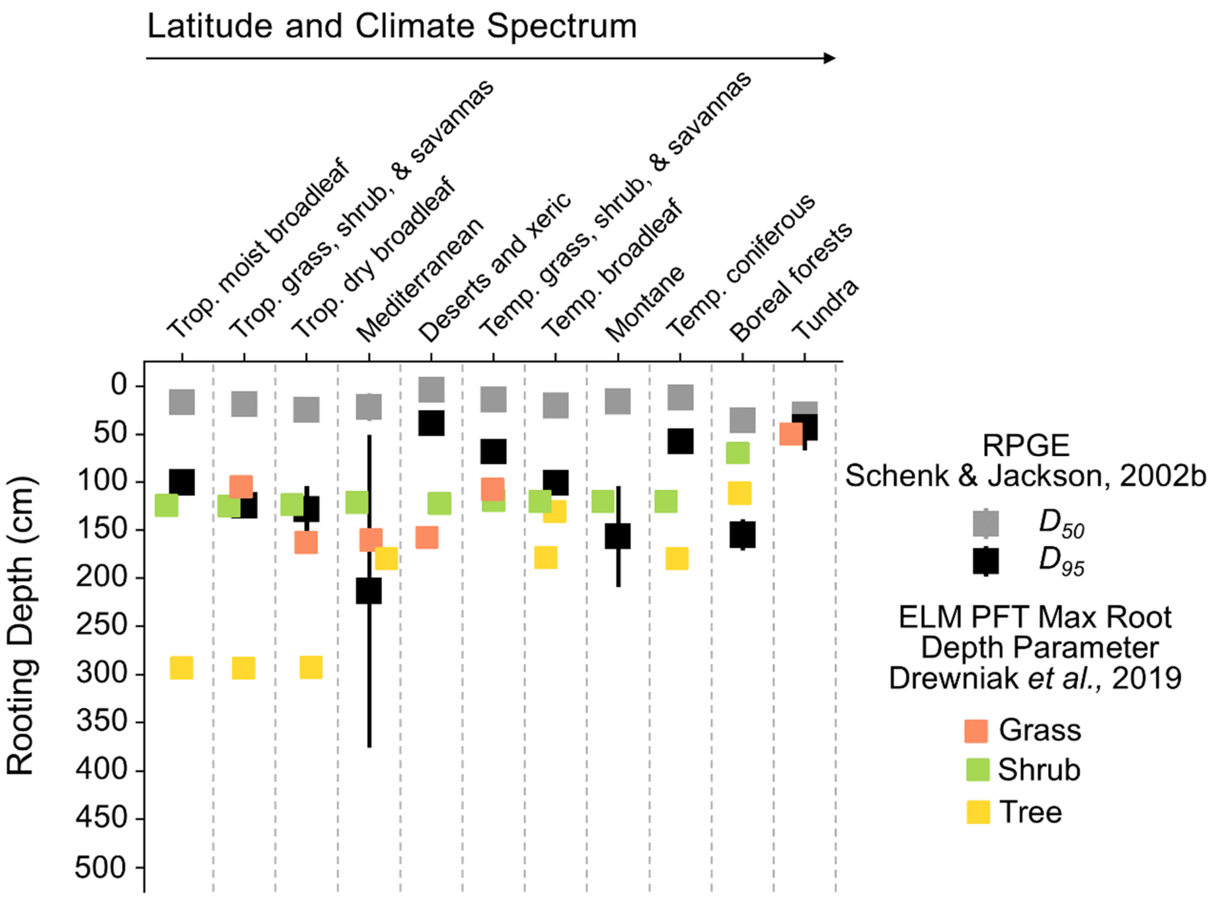

Fig. S2 Comparison of Root Profiles for Global Ecosystems (RPGE) D_{50} and D_{95} estimates (Schenk & Jackson, 2002b;2005) to the plant functional type (PFT) estimates used in the E3SM land model (ELM). Maximum Rooting Depth of PFTs in ELM were taken adapted from the values given in Table 1 from Drewniak *et al.* (2019). The error bars for the RPGE values represent the standard error. There are no error estimates for the ELM PFT max rooting depth values because they are singular values used as inputs for the model.

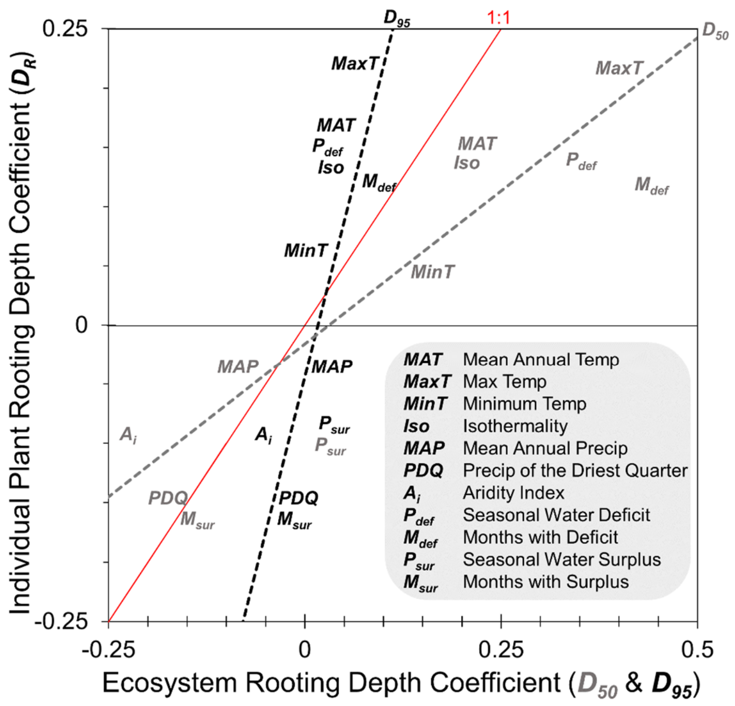

Fig. S3 Comparing the effect that climate variables had on individual plant rooting depth (RSIP D_R) on the y-axis to Root Profiles for Global Ecosystems (RPGE) D_{50} (gray) and D_{95} (black) on the x-axis. The coefficients were taken from linear mixed effects models with biome as random effects, rooting depth metrics as dependent variables, and climate parameters as fixed effects. Only a subset of the parameters used to measure the linear regressions are shown, with the labels defined in the figure key. The red line is the 1:1 baseline. The steeper black regression represents the relationship between D_R and D_{95}, where D_R is more sensitive to the climate metrics compared to D_{95} (y=2.62x - 0.04, R^2 = 0.71). The gray line with the lower slope represents the relationship between D_R and D_{50}, where D_{50} is more sensitive towards the climate metrics (y = 0.52x - 0.016, R^2 = 0.81). Coefficient values that fall near the 1:1 line indicate that both rooting depth estimates have similar relationships with the parameter. Variables included in the regression that were omitted from the plot were: Precipitation of Wettest Month, Precipitation of Driest Month, Precipitation of Wettest Quarter, Precipitation of Warmest Quarter, Precipitation of Coldest Quarter, Mean Temperature of Warmest Quarter, Mean Temperature of Coldest Quarter, Mean Diurnal Range, Temperature Seasonality, Temperature Annual Range, Mean Temperature of Wettest Quarter, and Mean Temperature of Driest Quarter.

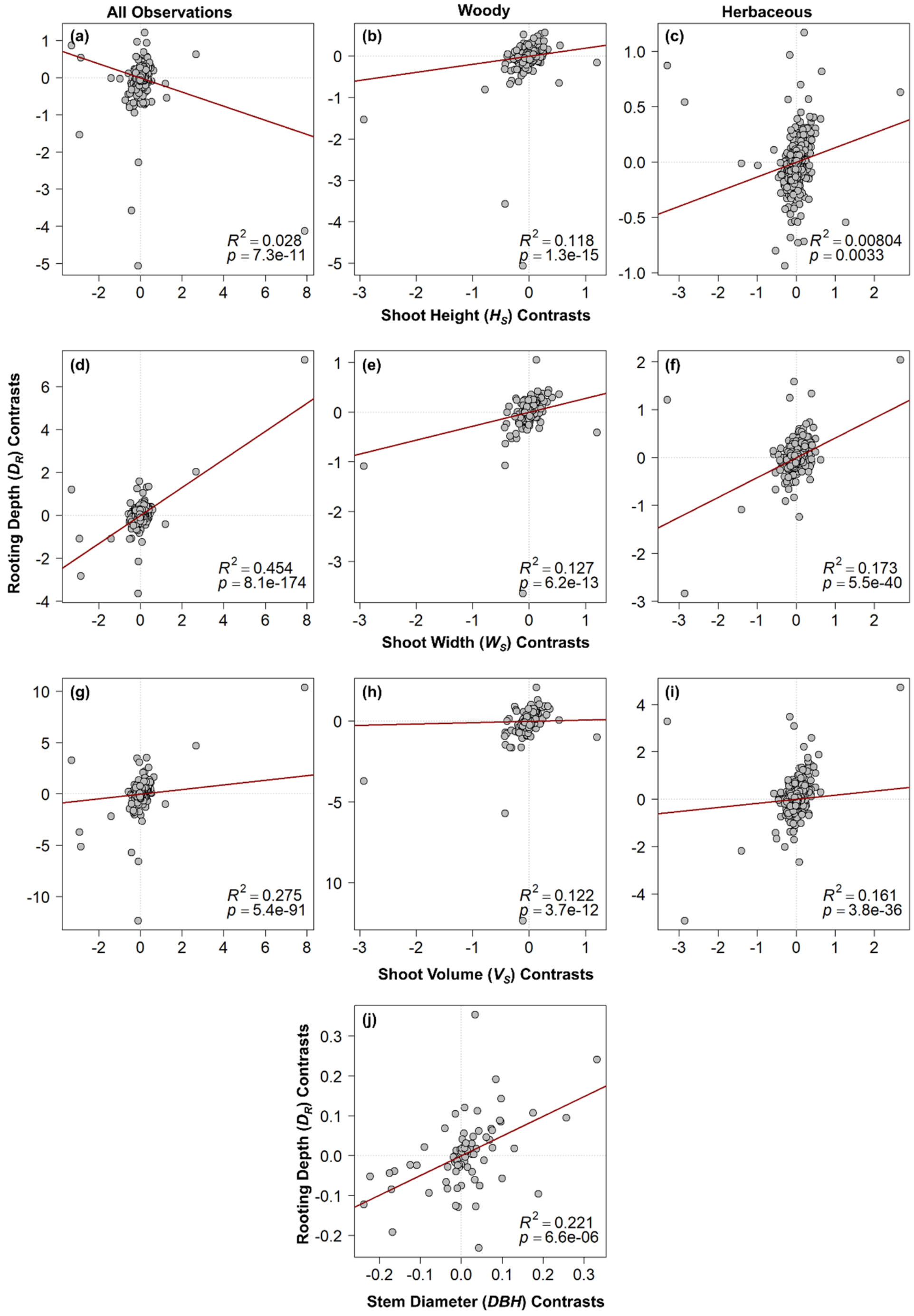

Fig. S4 The relationships between maximum rooting depth (D_R; y-axes) to aboveground plant size (H_S, W_S, V_S, & *DBH*; x-axes) analyzed using phylogenetically independent contrast regressions for all observations (a, d, & g), woody plants (b, e, h, & J), and herbaceous plants (c, f, & i). Statistically significant relationships ($p < 0.05$) are fitted with linear regressions using dark red lines.

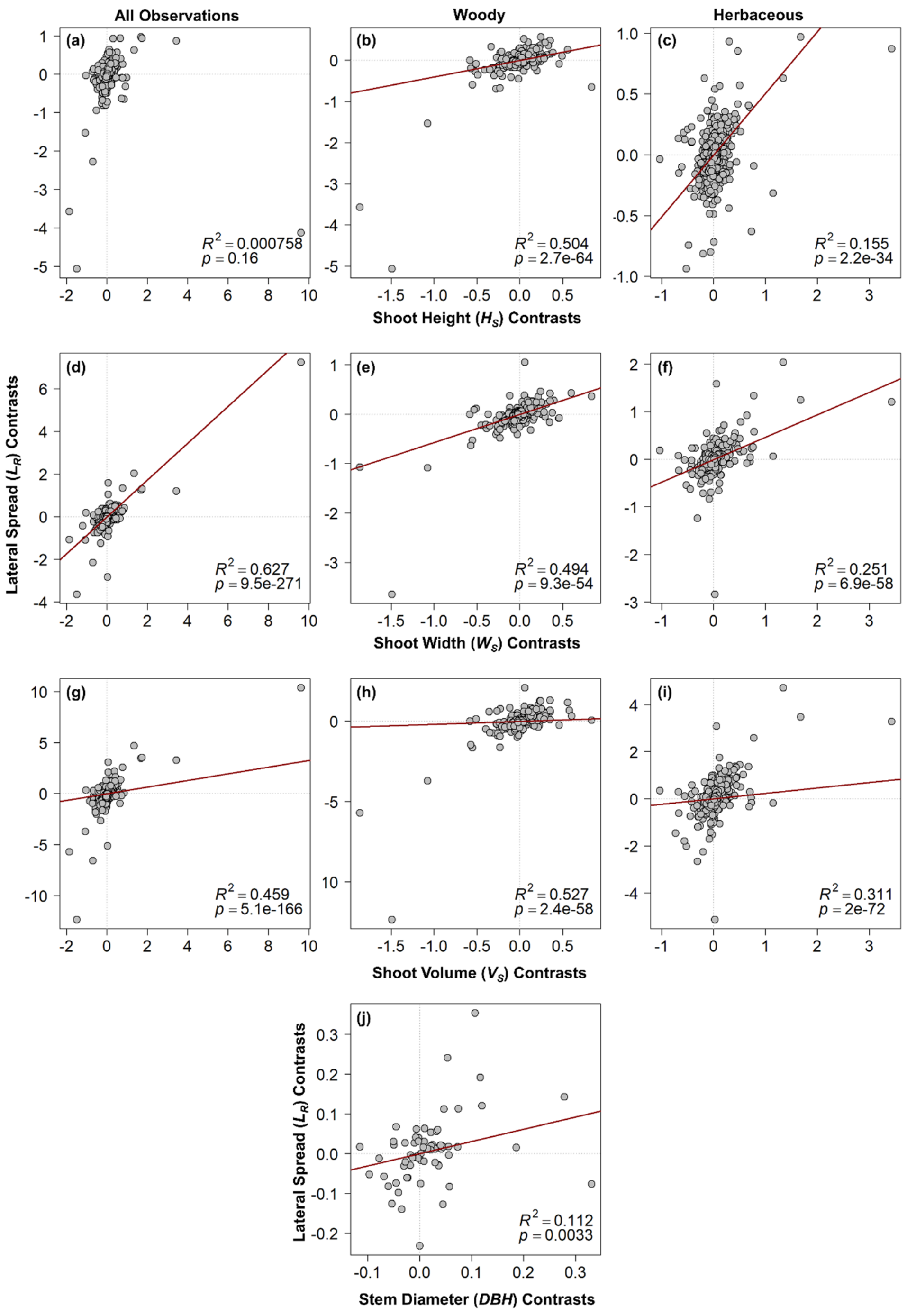

Fig. S5 The relationships between maximum lateral spread (L_R; y-axes) to aboveground plant size (H_S, W_S, V_S, & *DBH*; x-axes) analyzed using phylogenetically independent contrast regressions for all observations (a, d, & g), woody plants (b, e, h, & j), and herbaceous plants (c, f, & i). Statistically significant relationships ($p < 0.05$) are fitted with linear regressions using dark red lines.

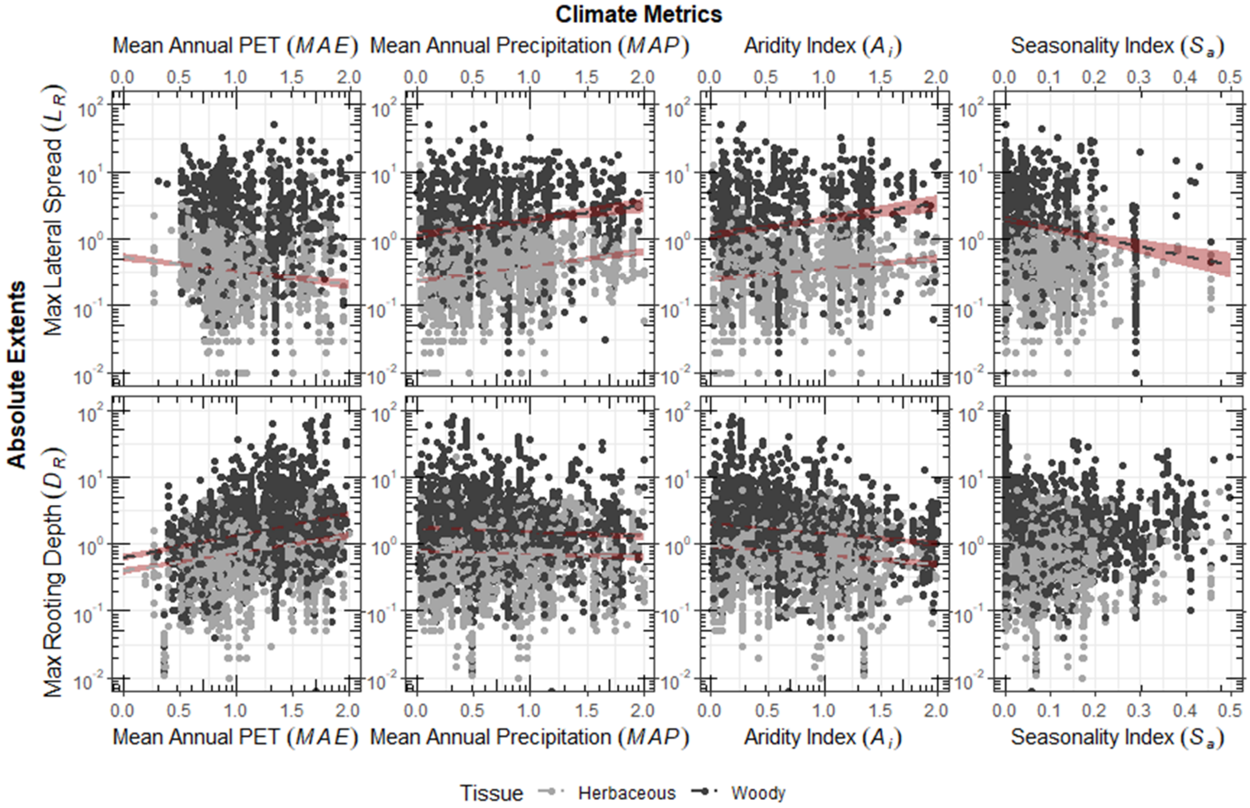

Fig. S6 The influence of climate metrics on max rooting depth (D_R; lower row) and max lateral spread *(*L_R; upper row). The climate metrics shown here, from left to right, are mean annual precipitation (*MAP*), mean annual potential evapotranspiration (*MAE*), mean annual precipitation (*MAP*), aridity index (A_i), seasonality index (S_a). Dark gray points are woody plants, and light gray points are herbaceous plants. The dashed lines (woody in dark gray and herbs in light gray) represent a linear regression where $p < 0.05$ in the form of $y = \beta + \alpha * x$, and the red shaded regions are the 95% confidence interval.The statistics and the parameters for the linear regressions are in Table **S4**. The axes scales are in common log (base 10).

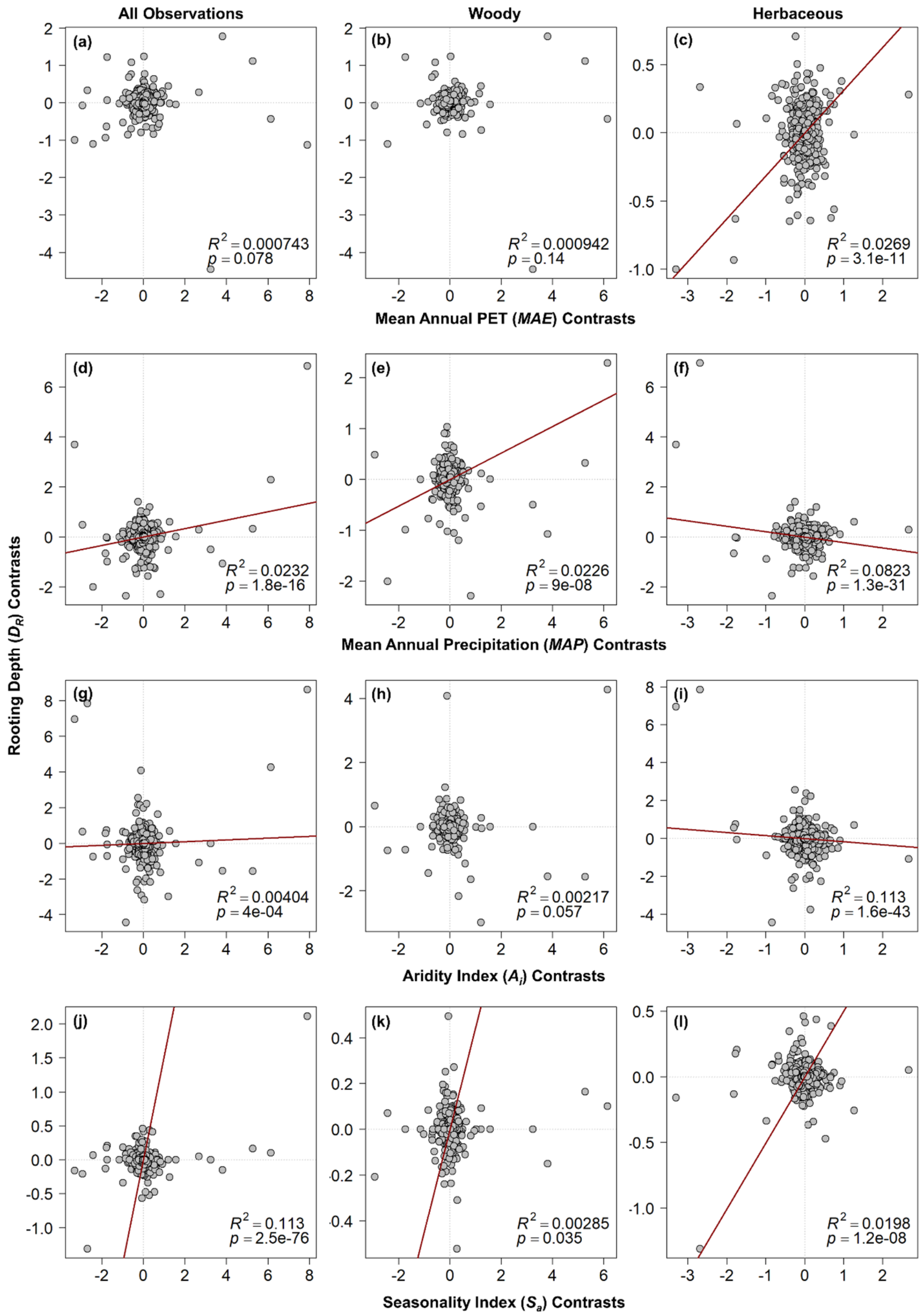

Fig. S7 The relationships between maximum rooting depth (D_R; y-axes) to the climate metrics (*MAE*, MAP, A_i, & S_a; x-axes) analyzed using phylogenetically independent contrast regressions for all observations (a, d, g, & j), woody plants (b, e, h, & k), and herbaceous plants (c, f, i, & l). Statistically significant relationships ($p < 0.05$) are fitted with linear regressions using dark red lines.

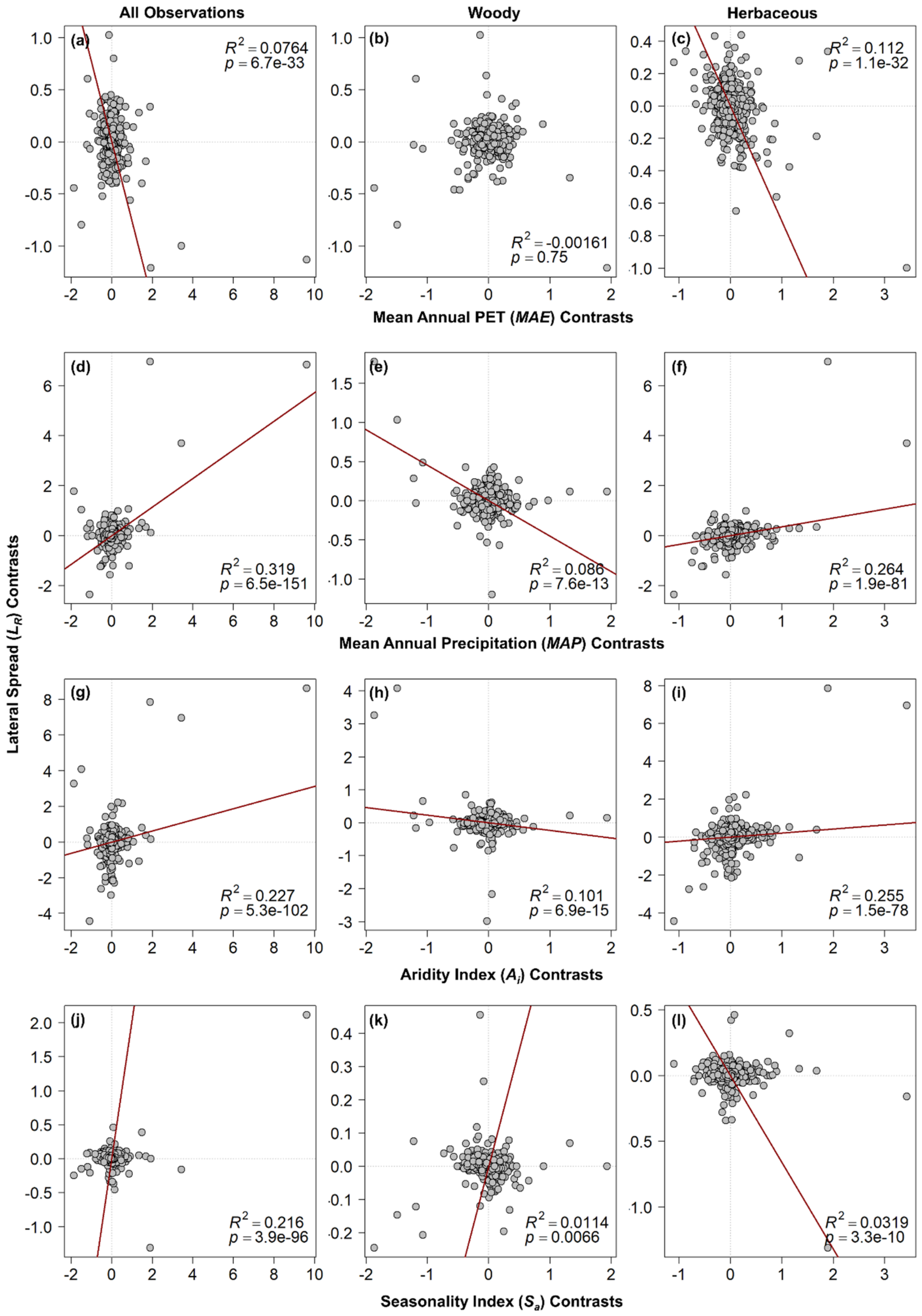

Fig. S8 The relationships between maximum lateral spread (L_R; y-axes) to the climate metrics (MAE, MAP, A_i, & S_a; x-axes) analyzed using phylogenetically independent contrast regressions for all observations (a, d, g, & j), woody plants (b, e, h, & k), and herbaceous plants (c, f, i, & l). Statistically significant relationships ($p < 0.05$) are fitted with linear regressions using dark red lines.

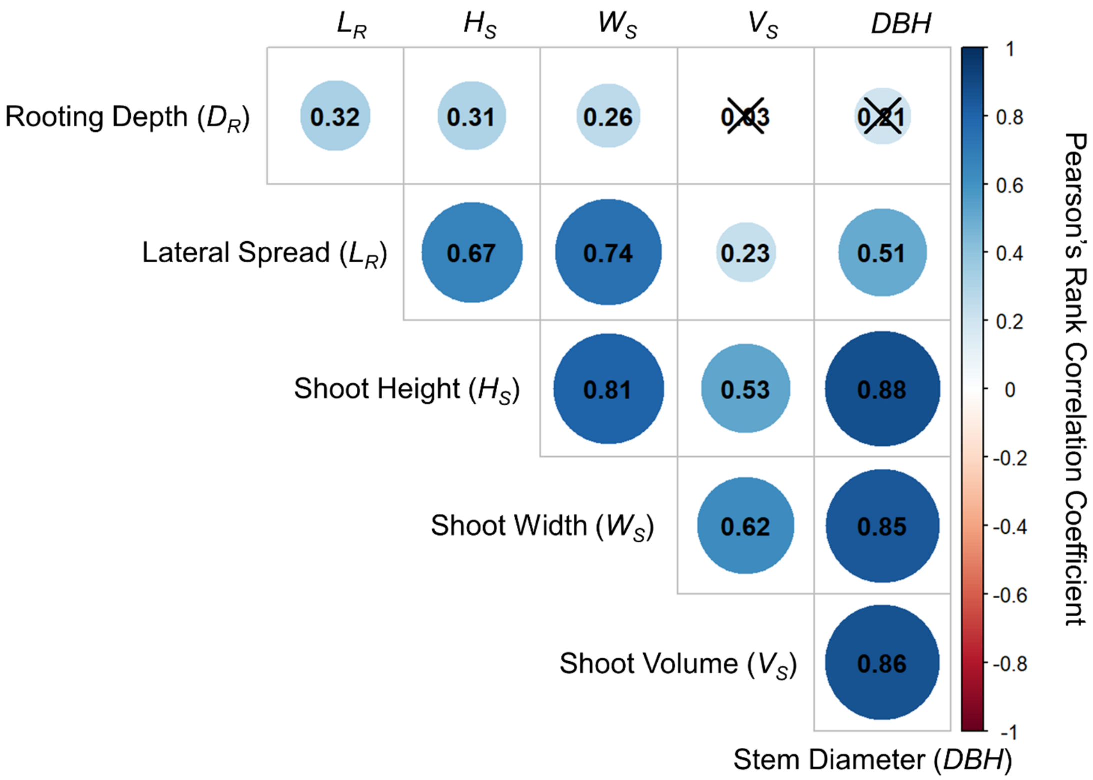

Fig. S9 Pearson's rank correlation matrix for the above- and belowground plant size metrics. Darker blue and larger circles represent more positive correlations. Insignificant correlations are crossed out ($p > 0.01$). Coefficient is written in black text.

Table S1 Description of RSIP Parameters (n is the total number of observations).

Entry identification			
Variable name	n	Description	Used in Random Forest?
ID	5647	Unique identification number for each observation	
Reference	5647	Reference that the entry data originates from, where it was taken from, and the figure/table/page #	
Plant description			
Variable name	n	Description	
Species	5639	current taxonomic nomenclature (*Genus species*). Found using plant databases (NRCS, 2021; Roskov et al., 2017; Smith & Brown, 2018)	
Family	5634	Current taxonomic family classification found using plant databases (NRCS, 2021; Roskov et al., 2017; Smith & Brown, 2018)	**X**
Life_span	5641	Plant life span found using plant databases (Roskov et al., 2017; NRCS, 2021) or reported in reference literature; (see Table **S2**)	**X**
Growth_form	5640	Plant growth form found using plant databases (Roskov et al., 2017; NRCS, 2021) or reported in reference literature (see Table **S2**)	**X**
Tissue	5997	Woody or herbaceous (see Table **S2**)	**X**
Seed_Cat	5630	Monocot, Dicot, or Gymnosperm (see Table **S2**)	**X**
Leaf_strategy	2515	Is the leafing strategy evergreen or deciduous (see Table **S2**)	**X**
Leaf_form	2206	Is the leafing strategy broad-leaf or needle-leaf (see Table **S2**)	**X**
PS_type	5649	Photosynthesis pathway (see Table **S2**)	**X**
Growth measurements			
Variable name	n	Description	
D_R	5633	Maximum rooting depth of plant [m]	**X**
L_R	2874	Maximum lateral root spread/one-sided (radius) linear distance from stem reached by roots [m]	**X**
W_R	1756	Rooting spread, aka maximum root system diameter [m]	
D_L	1769	Depth of maximum lateral root (L_R) [m]	
H_S	2373	Height of plant shoot [m]	**X**
W_S	2074	Width of plant shoot [m]	**X**
DBH	139	Diameter at breast height of trees branching higher than 1.5meters; recorded at 1.3-1.5 meters [cm]	

Relative measurements			
Variable name	n	Description	
V_R	2857	Estimate of root system volume using a bi-conal shape ($V_R[m^3] = \pi \times L_R^2 \times D_R \times 2/3$)	
V_S	2010	Estimate of shoot volume using an ellipsoid shape ($V_S [m^3] = \pi \times H_S[m] \times W_S[m]^2/6$)	
D_{rel}	2010	Relative maximum rooting depth ($D_{rel} = D_R / V_S$)	
L_{rel}	2010	Relative maximum lateral spread ($L_{rel} = L_R / V_S$)	
Dimensional aspect ratios and shoot:root ratios			
Variable name	n	Description	
$Y{:}X_{shoot}$	2010	Above-ground dimensional aspect ratio indicator ($Y{:}X_{shoot} = H_S/W_S$)	
$Y{:}X_{root}$	2938	Below-ground dimensional aspect ratio ($Y{:}X_{root} = D_R/W_R$)	
$S{:}R_y$	2368	Vertical coordinative strategy indicator ($S{:}R_y = H_S/D_R$)	
$S{:}R_x$	1974	Horizontal coordinative strategy indicator ($S{:}R_x = W_S/W_R$)	
Location			
Variable name	n	Description	
Biome	5609	Biome of entry, taken from original literature or based by location. following the WWF major habitat type classification (Olson *et al.*,2001; see **Table S2**)	**X**
Eco_Name	5559	Unique eco-regeion name (Olson *et al.*, 2001)	
Realm	5558	Biogeographic Realm (Olson *et al.*, 2001; see Table **S2**)	**X**
Eco_ID	5559	Unique id for the ecoregion with a realm-biome-ecoregion code in the form of: RR-BB-EE (Olson *et al.*, 2001)	**X**
Vegetation	2252	Dominant vegetation of ecosystem or biome (see Table **S2**)	
Location	5622	Geographic location (usually in terms of nearest city, state/province, region, or country)	
Lat	5591	Latitude (in decimal degrees)	**X**
Long	5591	Longitude (in decimal degrees)	**X**
Spatial_buffer	3332	Error estimate for the latitude and longitude, meant to serve as a buffer or radius that the point represents [km]	
Elevation	4327	Elevation [m]	**X**
Soil			
Variable name	n	Description	
Soil_description	4360	Description of soil	
ST_{USDA}	5191	Soil texture categories taken from the USDA system (see Table **S2**)	**X**
ST_{EU}	5189	Soil texture categories taken from the EU system (see Table **S2**)	**X**
Water_Table_Depth	507	Water Table Depth from Fan *et al.* (2017)	
Climate			
Variable name	n	Description	

BIO1	5559	Annual Mean Temperature [K]	**X**
BIO2	5559	Mean Diurnal Range (Mean of monthly (max temp - min temp)) [K]	**X**
BIO3	5559	Isothermality (BIO2/BIO7) (* 100)	**X**
BIO4	5559	Temperature Seasonality (standard deviation *100)	**X**
BIO5	5559	Max Temperature of Warmest Month [K]	**X**
BIO6	5559	Min Temperature of Coldest Month [K]	**X**
BIO7	5559	Temperature Annual Range (BIO5-BIO6) [K]	**X**
BIO8	5559	Mean Temperature of Wettest Quarter [K]	**X**
BIO9	5559	Mean Temperature of Driest Quarter [K]	**X**
BIO10	5559	Mean Temperature of Warmest Quarter [K]	**X**
BIO11	5559	Mean Temperature of Coldest Quarter [K]	**X**
BIO13	5559	Precipitation of Wettest Month [m]	**X**
BIO14	5559	Precipitation of Driest Month [m]	**X**
BIO15	5559	Precipitation Seasonality (Coefficient of Variation)	**X**
BIO16	5559	Precipitation of Wettest Quarter [m]	**X**
BIO17	5559	Precipitation of Driest Quarter [m]	**X**
BIO18	5559	Precipitation of Warmest Quarter [m]	**X**
BIO19	5559	Precipitation of Coldest Quarter [m]	**X**
MAP	5590	Mean annual precipitation. taken from primary literature or WorldClim data (BIO12) [m]	**X**
MAE	5587	Mean annual potential evapotranspiration. taken from primary literature or calculated using Hargreave's equation (Trabucco & Zomer, 2019)	**X**
A_i	5587	Aridity index (=MAP/MAE)	**X**
S_a	5553	Annual water storage index: $S_a = min[P_{sur}, P_{def}]$	**X**
M_{sur}	5493	Months with a surplus of water (MAP_m - PET_m > 0)	**X**
M_{def}	5552	Months with a deficit of water (PET_m - MAP_m > 0)	**X**
P_{sur}	5493	Seasonal surplus of water: $P_{sur} = \sum_{m,\ MAP_m - PET_m > 0} (MAP_m - PET_m)$	**X**
P_{def}	5552	Seasonal deficit of water: $P_{def} = \sum_{m,\ PET_m - MAP_m > 0} (PET_m - MAP_m)$	**X**
R_{class}	5590	Classification of MAP (see Table **S2**)	**X**
R_{regime}	5615	Precipitation seasonality regime classified using MAP, latitude, and BIO18-19 (see Table **S2**)	**X**
A_{class}	5587	Classification of A_i (see Table **S2**)	**X**

Table S2 RSIP categorical groups. The number of total observations (n), and unique species, geographic locations, and studies for each class is shown.

Life_span					
Class	n	species	locations	studies	Description
A	557	367	198	82	Annual plants (whole life cycle within 1 year)
P	5082	2635	1941	611	Perennial plants (includes biennials and all non-annual plants)
Growth_Form					
Class	n	species	locations	studies	Description
Forb	1715	1207	528	116	Forbs, herbaceous plants that are not grasses
Grass	991	467	374	158	Grasses
Semi-shrub	574	318	206	111	Semi-shrub
Shrub	694	367	351	194	Shrub
Succulent	89	67	54	49	Succulent
Tree	1576	613	1010	350	Tree
Tissue					
Class	n	species	locations	studies	Description
Herbaceous	2706	1674	764	211	Herbaceous plants
Woody	2844	1264	1427	530	Woody plants
Seed_Cat					
Class	n	species	locations	studies	Description
Dicot	3947	2255	1514	506	Dicot Plants (Magnoliopsida)
Gymnosperm	523	136	340	118	Gymnosperm Plants
Monocot	1135	577	420	170	Plants with only one cotyledon
Pteridophyte	24	20	19	16	Pteridophyte plants
Leaf_strategy					
Class	n	species	locations	studies	Description
D	1130	467	721	265	Deciduous (including winter-deciduous and drought-deciduous)
E	1385	602	126	363	Evergreen (including some semi-evergreen)
Leaf_form					
Class	n	species	locations	studies	Description
B	1640	780	967	403	Broad-leaf
N	557	163	352	127	Needle-leaf
P	9	8	9	8	Palm
PS_type					
Class	n	species	locations	studies	Description
C3	4918	2660	1899	583	C3 Photosynthetic Pathway
C3-C4	617	255	261	138	C3/C4 intermediates

C4	10	4	7	6	C4 Photosynthetic Pathway
CAM	103	75	59	52	Crassulacean Acid Metabolism Photosynthesis
Biome					
Class	n	species	locations	studies	Description
1	1240	731	306	191	Deserts & xeric shrublands
2	144	106	75	47	Tropical & subtropical moist broadleaf forests
3	247	128	222	22	Tropical & subtropical dry broadleaf forests
4	3	3	3	2	Tropical & subtropical coniferous forests
5	649	416	324	103	Temperate broadleaf & mixed forests
6	1111	706	383	43	Temperate Conifer Forests
7	187	50	108	26	Boreal forests/Taiga
8	301	165	103	63	Tropical & Subtropical Grasslands, Savannas & Shrublands
9	1020	552	350	86	Temperate Grasslands, Savannas & Shrublands
10	65	59	26	9	Flooded Grasslands & Savannas
11	222	171	25	15	Montane Grasslands & Shrublands
12	25	21	6	4	Tundra
13	379	253	142	73	Mediterranean Forests, Woodlands & Scrub
Realm					
Class	n	species	locations	studies	Description
AA	205	137	67	36	Australasia
AT	512	270	315	85	Afrotropical
IM	143	97	36	30	Indo-Malayan
NA	1803	803	733	235	Nearctic
NT	333	218	105	66	Neotropical
OC	1	1	1	1	Oceania
PA	2560	1541	757	154	Palearctic
Vegetation					
Class	n	species	locations	studies	Description
G	804	527	120	61	Grasses, Grassland
S	1024	642	160	120	Shrubs, Shrubland
T	423	249	124	91	Trees, Forested
USDA_soil_texture					
Class	n	species	locations	studies	Description
Cl	222	177	105	61	Clay
SiCl	33	27	19	14	Silty clay

SaCl	201	108	96	37	Sandy clay
ClLo	248	221	107	53	Clay loam
SiClLo	74	51	45	23	Silty clay loam
SaClLo	97	84	64	33	Sandy clay loam
Lo	897	616	248	86	Loam
SiLo	366	251	181	60	Silty loam
SaLo	798	539	302	116	Sandy loam
Si	115	89	75	23	Silt
LoSa	588	401	261	62	Loamy sand
Sa	1205	821	496	225	Sand
Wa	126	104	55	23	Wetland
Ro	212	160	110	44	Rocky
EU_soil_texture					
Class	n	species	locations	studies	Description
F	704	499	316	289	Fine
M	1442	911	522	544	Medium
C	2916	1779	1152	1400	Coarse
Rclass					
Class	n	species	locations	studies	Description
A	281	201	80	56	MAP ≤ 0.125
B	710	434	164	103	0.125 < MAP ≤ 0.25
C	1154	695	319	142	0.25 < MAP ≤ 0.5
D	3444	1963	1469	364	MAP > 0.5
Rregime					
Class	n	species	locations	studies	Description
E	704	455	207	104	all year; climates where 0.75 < BIO18/BIO19 < 1.25
S	3140	1690	1090	235	summer; temperate and subtropical climates where BIO18/BIO19 ≥1.25
TS	711	395	420	142	tropical seasonal; seasonally dry climates lacking a cold season
W	1059	690	336	182	winter; temperate and subtropical climates where BIO18/BIO19 ≤0.75
Aclass					
Class	n	species	locations	studies	Description
ha	76	65	25	20	hyper-arid; AI < 0.03
ar	882	530	233	142	arid; 0.03 ≤AI < 0.2
sa	1346	778	459	164	semi-arid; 0.2 ≤AI < 0.5
sh	595	406	265	80	sub-humid; 0.5 ≤AI < 0.65
hu	2687	1599	1054	277	humid; AI ≥0.65

Table S3 Pagel's Lambda values for the above- and belowground plant measurements with the calculated log likelihood statistic (logL).

Trait	Lambda (λ)	logL
**Rooting Depth (D_R)**		
All Observations	0.553	-7872
Woody	0.271	-3835
Herbaceous	0.644	-2098
**Lateral Spread (L_R)**		
All Observations	0.968	-4648
Woody	0.865	-1811
Herbaceous	0.186	-1322
**Shoot Height (H_S)**		
All Observations	0.985	-3937
Woody	0.934	-1664
Herbaceous	0.558	-269
**Shoot Width (W_S)**		
All Observations	0.0001	-7784
Woody	0.750	-1010
Herbaceous	0.0001	-5621
**Shoot Volume (V_S)**		
All Observations	0.0001	-15824
Woody	1.000	-3423
Herbaceous	0.0001	-11252
**Stem Diameter (*DBH*)**		
Trees	0.922	-349

*Lambda is color coded from low to high phylogenetic signal: low lambda (< 0.3) in white, moderate lambda (0.3 to 0.6) in light blue, high lambda (0.6 to 0.9) in medium blue, very high lambda (> 0.9) in darker blue with white text.

Table S4 Linear and phylogenetically independent contrast (PIC) relationships of belowground extents (D_R & L_R) to climate metrics (*MAE*, *MAP*, A_i, & S_a) in the form of $y=\beta_0 + \beta_1 x$, where y is D_R or L_R, where β_0 is the intercept (Int.) and β_1 is the slope. PIC regression intercepts set to zero.

	Max. Rooting Depth (D_R)					Max. Lateral Spread (L_R)				
	Linear Regression			PIC		Linear Regression			PIC	
	Int.	*Slope (SE)*	R^2 *& p*	*Slope (SE)*	R^2 *& p*	*Int.*	*Slope (SE)*	R^2 *& p*	*Slope (SE)*	R^2 *& p*
**Mean Annual PET (MAE)**										
All Observations	-0.4	0.4 (0.016)	0.1***	-0.067 (0.038)	0.001	-0.4	0.16 (0.034)	0.007***	-0.77 (0.063)	0.076***
Woody	-0.2	0.33 (0.021)	0.081***	-0.076 (0.052)	0.001	0.25	-0.042 (0.053)	0.001	-0.023 (0.075)	-0.002
Herbaceous	-0.4	0.25 (0.026)	0.035***	0.31 (0.047)	0.027***	-0.3	-0.21 (0.034)	0.021***	-0.71 (0.058)	0.112***
**Mean Annual Precipitation (MAP)**										
All Observations	0.07	-0.067 (0.012)	0.006***	0.17 (0.020)	0.023***	-0.3	0.12 (0.024)	0.009***	0.57 (0.020)	0.319***
Woody	0.26	-0.096 (0.015)	0.015***	0.26 (0.049)	0.023***	0.06	0.21 (0.037)	0.029***	-0.45 (0.062)	0.086***
Herbaceous	-0.1	-0.053 (0.018)	0.003*	-0.22 (0.018)	0.082***	-0.6	0.17 (0.021)	0.037***	0.35 (0.017)	0.264***
**Aridity Index (Ai)**										
All Observations	0.14	-0.15 (0.0089)	0.047***	0.050 (0.014)	0.004**	-0.3	0.063 (0.014)	0.007***	0.31 (0.014)	0.227***
Woody	0.31	-0.17 (0.013)	0.062***	0.066 (0.035)	0.002	0.09	0.15 (0.025)	0.031***	-0.23 (0.029)	0.101***
Herbaceous	-0.1	-0.094 (0.011)	0.026***	-0.16 (0.011)	0.113***	-0.6	0.1 (0.012)	0.043***	0.21 (0.10)	0.255***
**Seasonality Index (Sa)**										
All Observations	0.02	0.068 (0.05)	0	1.54 (0.081)	0.113***	-0.2	-0.63 (0.12)	0.01***	2.0 (0.092)	0.216***
Woody	0.18	-0.018 (0.06)	0	0.45 (0.21)	0.003†	0.28	-1.1 (0.19)	0.031***	0.71 (0.26)	0.011*
Herbaceous	-0.1	-0.078 (0.079)	0	0.50 (0.088)	0.02***	-0.5	0.14 (0.1)	0.001	-0.66 (0.10)	0.032***

P*-value significance codes: * < 0.0001; ** < 0.001; * < 0.01; † <0.05

Table S5 Nonlinear regression curves for the shape ratios plotted on Fig. **7.**

	Formula	R^2	*Curves Intercept*
**Woody**			
$Y{:}X_{root}$	$= 1.5*exp(-0.5*(ln(A_i/0.26)/1.2)^2)$	0.88	x=0.44, y=1.34
$Y{:}X_{shoot}$	$= 1.7*A_i/(0.1 + A_i)$	0.85	
$S{:}R_y$	$= 14*A_i/(4.4 + A_i)$	0.87	x=0.14, y=0.43
$S{:}R_x$	$= 0.58*exp(-0.5*(ln(A_i/0.43)/1.5)^2)$	0.34	
**Herbaceous**			
$Y{:}X_{root}$	$= 1.7*x/(-0.005 + A_i)$	0.23	x=0.10, y=1.96
$Y{:}X_{shoot}$	$= 1.3*x/(-0.04 + A_i)$	0.04	
$S{:}R_y$	$= 1.7*x/(-0.005 + A_i)$	0.67	x=0.18, y=0.55
$S{:}R_x$	$= 0.42*x/(-0.04 + A_i)$	0.85	

References

Drewniak BA. **2019**. Simulating Dynamic Roots in the Energy Exascale Earth System Land Model. *Journal of Advances in Modeling Earth Systems* **11**: 338–359.

Fan Y, Miguez-Macho G, Jobbágy EG, Jackson RB, Otero-Casal C. **2017**. Hydrologic regulation of plant rooting depth. *Proceedings of the National Academy of Sciences* **114**: 10572.

Fick SE, Hijmans HJ. **2017**. Worldclim 2: New 1-km spatial resolution climate surfaces for global land areas. *Int. J. Climatol*. **37**: 4302-4315.

Olson DM, Dinerstein E, Wikramanayake ED, Burgess ND, Powell GVN, Underwood EC, D'amico JA, Itoua I, Strand HE, Morrison JC, et al. 2001. Terrestrial Ecoregions of the World: A New Map of Life on Earth: A new global map of terrestrial ecoregions provides an innovative tool for conserving biodiversity. *BioScience* **51**: 933–938.

Roskov Y, Abucay L, Orrell T, Nicolson D, Bailly N, Kirk PM, Bourgoin T, DeWalt RE, Decock W, De Wever A, Nieukerken E van, Zarucchi J, Penev L, eds. 2017. *Species 2000 & ITIS Catalogue of Life, 2017 Annual Checklist*. Digital resource at www.catalogueoflife.org/annual-checklist/2017. *Species 2000: Naturalis, Leiden*, the Netherlands. **ISSN 2405-884X.** [accessed 1 September 2020].

Schenk HJ, Jackson RB. **2002a**. Rooting depths, lateral root spreads and below-ground/above-ground allometries of plants in water-limited ecosystems. *Journal of Ecology* **90**: 480–494.

Schenk HJ, Jackson RB. **2002b**. The global biogeography of roots. *Ecological Monographs* **72**: 311–328.

Schenk HJ, Jackson RB. **2005**. Mapping the global distribution of deep roots in relation to climate and soil characteristics. *Geoderma* **126**: 129–140.

Smith SA, Brown JW. 2018. Constructing a broadly inclusive seed plant phylogeny. *American Journal of Botany* **105**: 302-314.

Trabucco A, Zomer RJ. **2019**. *Global Aridity Index and Potential Evapo-Transpiration (ET0) Climate Database v2*. figshare. Fileset. https://doi.org/10.6084/m9.figshare.7504448.v3. [accessed 10 September, 2020]

USDA, NRCS. 2021. The PLANTS Database (http://plants.usda.gov). *National Plant Data Team*, Greensboro, NC USA. [accessed 10 September 2021].
